# Uncertain Multiplicative Language Decision Method Based on Group Compromise Framework for Evaluation of Mobile Medical APPs in China

**DOI:** 10.3390/ijerph17082858

**Published:** 2020-04-21

**Authors:** Junchang Li, Jiantong Zhang, Ye Ding

**Affiliations:** School of Economics and Management, Tongji University, Shanghai 200092, China; junchangli_sem@tongji.edu.cn (J.L.); zhangjiantong@tongji.edu.cn (J.Z.)

**Keywords:** M-medical service, evaluation of APP, uncertain multiplicative linguistic variable, group compromise ranking

## Abstract

The mobile medical application (M-medical APP) can optimize medical service process and reduce health management costs for users, which has become an important complementary form of traditional medical services. To assist users including patients choose the ideal M-medical APP, we proposed a novel multiple attribute group decision making algorithm based on group compromise framework, which need not determine the weight of decision-maker. The algorithm utilized an uncertain multiplicative linguistic variable to measure the individual original preference to express the real evaluation information as much as possible. The attribute weight was calculated by maximizing the differences among alternatives. It determined the individual alternatives ranking according to the net flow of each alternative. By solved the 0–1 optimal model with the objective of minimizing the differences between individual ranking, the ultimate group compromise ranking was obtained. Then we took 10 well-known M-medical APPs in Chinese as an example, we summarized service categories provided for users and constructed the assessment system consisting of 8 indexes considering the service quality users are concerned with. Finally, the effectiveness and superiority of the proposed method and the consistency of ranking results were verified, through comparing the group ranking results of 3 similar algorithms. The experiments show that group compromise ranking is sensitive to attribute weight.

## 1. Introduction

Medical is the basic industry of the country, which is closely related to the health and development of the whole country. The prominent characteristics of the medical industry are strong professionalism and asymmetric information. In recent years, residents have valued healthcare and quality of life due to the disposable income of residents increasing. The degree of population aging has been further deepened in many countries. More convenient and quality medical services are urgently needed for the older generation, who are more vulnerable to disease and likely to contact with the doctor directly. Simultaneously, serious public health incidents occur from time to time, which put great pressure on the normal operation of the medical system, even bring catastrophic damage. Under the influence of these factors, there are still a series of problems in China’s medical care system, such as the imbalance of regional medical resources, the tension between doctors and patients and the low efficiency of medical services. To deal with these problems, the Chinese government has continued to deepen reform and issued programs in the medical sector, for example, medical big data, medical informatics, “Internet + medical” and healthy China 2020, which have made achievements in the transformation and upgrading of the medical industry.

**Figure 1:**
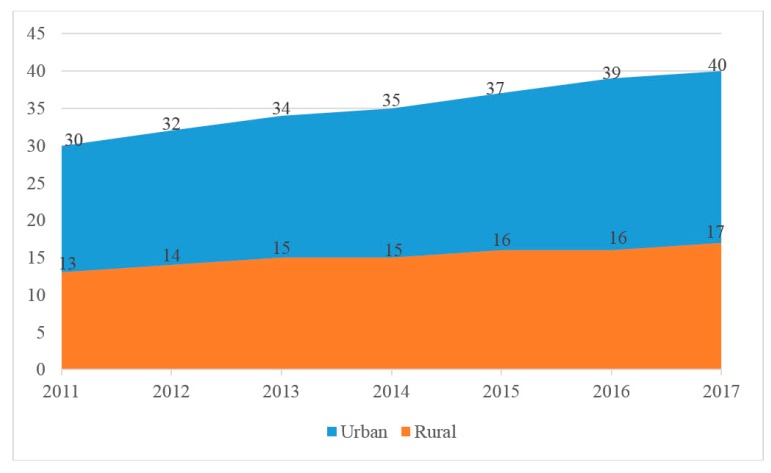
Number of licensed doctors in urban and rural areas of China from 2011 to 2017. Data from China Bureau of Statistics.

In the past six years, medical resources in urban and rural areas have been expanding. However, the expansion speed of urban medical resources is faster, and the imbalance between urban and rural medical resources is further aggravated. Taking the number of licensed doctors or assistant doctors per 10k people as an example, Figure 1 shows the change of quantity from 2011 to 2017. The gap between urban and rural areas was 17 in 2011 and 23 in 2017. Consequently, it’s necessary to further reform the existing supply structure and business model in China’s medical market, especially in the defense of public health emergency, most of suspected patients cannot get timely diagnosis and treatment in the countryside. Furthermore, the problem of unequal medical resources as a global issue is not only in China but also in other countries. In the United States, with only 14% of practicing primary care physicians providing services to 25% of the rural residents [1].

**Figure 2:**
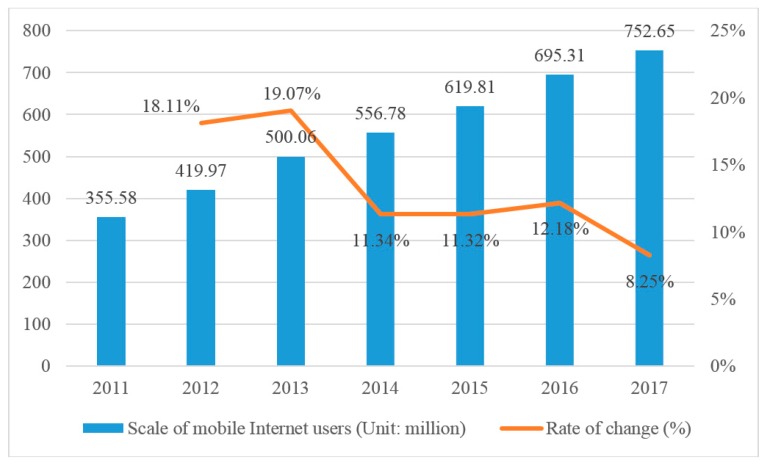
The change of mobile Internet users from 2011 to 2017 in China. Data from China Internet Network Information Center.

Mobile Internet highlights the advantages of Internet anytime, anywhere, convenient and fast. With the development and maturity of mobile information technology and popularization of intelligent mobile terminal, the scale of mobile Internet users has increased year by year in China, which is presented in Figure 2. It shows that by the end of 2017, the number of mobile Internet users was 752.65 million, and increased by 8.25% compared with 2016 in China.

**Figure 3:**
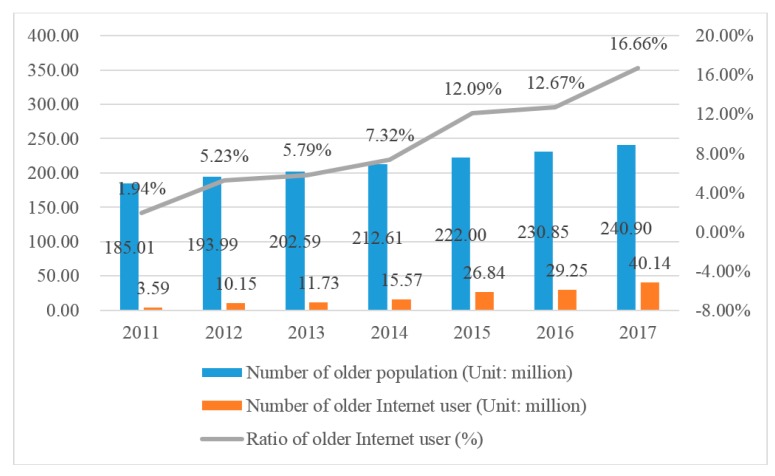
The popularity rate of Internet in Chinese older population from 2011 to 2017. Data from China Bureau of Statistics and Internet Network Information Center.

The elderly (age 60+) ask for medical services more frequently and tend to have high-quality medical resources in daily life. Due to the aging of body function, the older population often suffer from one or more chronic diseases and face a health emergency. As Figure 3 shows, there is a growth trend on the number of elderly from 2011 to 2017 in China, in which the number of older Internet users is increasing as well. Fortunately, the growth rate of older Internet users is higher than that of older population. Correspondingly, the popularity rate of Internet in Chinese older population was 16.66% in 2017 and greater than 12.67% in 2016. It indicates that the medical service that is delivered by mobile Internet is gradually known and accepted by the Chinese older generation and has great potential for development in the future.

Practically, medical resources are tight and a large number of patients gather together when public health emergencies occur, which leads to a vicious circle of the medical system. In order to maintain the normal operation of the whole medical system, medical institutions move some dangerous medical services online. At the same time, they trust the simple and mechanical medical process in the mobile medical applications (M-medical APPs), so as to improve the efficiency of medical services. The advantages of remote diagnosis and Internet hospital have been proved in practice. These Internet-based applications not only reduce cross infection between medical staff and patients, but also balance medical resources.

With the penetration of mobile Internet in rural areas of China, mobile information technology empowers the medical industry, which may be able to effectively solve the contradictions and problems in the medical field [2], and then improve the medical service level of the whole country. For instance, Alihealth realized cross-regional sharing and allocation of existing social medical and health resources. In this climate, practitioners and researchers pay more and more attention to the changes and upgrades of mobile information technologies in the area of medical care. The practice of using mobile technology infrastructure in medical care, especially 5G and smartphone, is termed as Mobile Medical [3,4]. Mobile Medical can improve the quality, safety and efficiency in current healthcare, and provides several new medical services with ubiquitous and mobile devices [2]. Additionally, it has greatly improved the convenience of patients’ medical treatment and drug purchases to meet consumers’ pursuit of a healthy lifestyle. The mobile devices include smartphones, tablets, PDA and laptop computers in common, which are installed Android, Apple or Windows operating systems. Many types M-medical APPs are developed to assist various target groups accessing high-quality medical services anywhere and anytime, since the features of mobile devices and technologies.

M-medical APPs are increasingly important for the health industry [5]. The existence of mobile integrated medical care and community paramedicine programs is reasonable and beneficial to the improvement of local medical and health environments [1]. Mobile medical care has a broad application prospect [6]. Although China’s mobile medical industry is still in its infancy [2], medical providers, technology companies, Internet enterprises, information platforms, etc. have developed mobile medical business and launched unique M-medical APPs. An increasing number of M-medical APPs are developed benefiting health service delivery [2]. As of December 2019, 20 M-medical APPs are active in Apple store in China market. Nevertheless, users encounter difficulty in choosing the appropriate APP when facing the abundant number of applications, because they could not invest an amount of time in determining APPs, and the market is full of commercial information to promote APPs.

However, nowadays people’s daily life has been flooded with numerous mobile applications [7]. In regards to M-medical APP, which APP should ordinary users choose to gain better medical experience? Which performance of mobile medical application should be designed or improved so as to be accepted by the public, for mobile medical companies? Unfortunately, there are few published studies on the evaluation of M-medical APPs, let alone that of China. The previous research around M-medical APP focuses on the following areas: (a) the development or improvement of M-medical APP [2,4,8,9,10]; (b) the attitudes of different groups to mobile medical technology or APP, including technical and medical students, working staff and healthcare professor [2]; (c) classification of M-medical APP and macro analysis of mobile medical industry [11,12]; (d) design or development of specific M-medical APP, such as in oncology [13], in Neurosurgery [14] and in chronic diseases [15]. Therefore, there is a pressing need for devising ways to evaluate the available M-medical APPs effectively.

To fill the gaps, the current study develops the uncertain multiplicative linguistic variable decision-making algorithm based on the existing group decision-making framework. After analyzing the medical services provided by app, the algorithm is utilized to investigate the expert group’s evaluation of M-medical APPs in China. The purpose of the paper includes providing mobile medical service providers and researchers with macro-understanding, and help users select the most appropriate APP. The structure of the paper is arranged as follows. In Section 2, works of literature related to M-medical APPs and evaluation of mobile medical healthcare application are reviewed. Section 3 summarizes the services provided by M-medical APPs. In Section 4, some basic concepts about linguistic terms are introduced. Section 5 designs the algorithm that uncertain multiplicative linguistic decision method based on group compromise ranking framework and analyze the characteristic of the algorithm. Section 6 constructs the assessment system and ranks 10 M-medical APPs using the proposed method. In Section 7, the comparative analysis of 3 similar algorithms is carried out. In Section 8, some conclusions are presented and further studies are discussed.

## 2. Related Literature

At present, the unified concept of mobile medical application has not been formed yet. Few studies carried out into the aforementioned four areas are as follows: Mark et al. developed the mobile traditional Chinese medicine application of the Android system using mobile devices and cloud services, which improved service efficiency and patient safety [16]. Katz and Rice investigated the attitudes of American adults toward mobile medical technology [6]. Vishnu et al. compared the diagnostic accuracy of the APP against neurology residents in movement disorders and asserted M-medical APP can effectively help doctors diagnose illness [17]. Oluwagbemi et al. developed a mobile application for some hereditary diseases and disorders, and compared the performance of similar APPs through analysis online and offline platform questionnaires [18]. Gabor et al. built a mobile APP that can diagnose users’ diseases. The diagnosis process is divided into two stages: forming a preliminary medical diagnosis by analyzing a series of questions answered by users; providing users with the opportunity to communicate with medical experts for accurate medical diagnosis [19].

On the topic of evaluating/assessing/ranking/ordering M-medical APPs, very limited studies were searched in archived literature. Thus, we reviewed similar research related to mobile healthcare application, due to mobile healthcare contains mobile medical, which has always been concerned with the wave of emerging technology. Mobile Application Rating Scale is often used to evaluate mobile health APP in some healthcare subdivision domains. The evaluation system consists of five dimensions: engagement, functionality, aesthetics, information and subjective quality. Furthermore, each dimension is subdivided into several sub-indicators such as interactivity, performance, ease of use, aesthetics, accuracy, quality, quantity, credibility and evidence based [20]. Some researchers have integrated the Fuzzy Analytic Hierarchy Process (FAHP) and Technique for Order of Preference by Similarity to Ideal Solution (TOPSIS) into a decision-making approach for ranking alternatives from multiple attributes. In the approach, fuzzy AHP and TOPSIS were utilized to calculate the weight of evaluation attributes and ultimately ranking respectively [21]. Rajak and Shaw suggested a Fuzzy Multiple Attributes Decision Making (FMADM) methodologies, consisting of AHP and fuzzy TOPSIS, for accessing and selecting M-medical APPs. An evaluation structure was constructed considering nine main attributes, i.e., user satisfaction, compatibility, functionality, security, accessibility, easy to learn and use, empathy, information quality, responsiveness [7]. Chen developed a model, namely fuzzy geometric mean-FAHP, to assess the importance of critical factors influencing the application of smart technology to mobile health care [22]. Through interviews, observation, surveys and exams, Hao et al. collected the data of mobile English vocabulary learning APP, and students’ evaluation information [23].

Evaluation is one of the essential parts for decision-making. The purpose of the evaluation is to make scientific decisions. In prior studies, decision-makers were forced to evaluate with real numbers, which limited their cognitive thinking process to a specific range, and more likely to have inaccurate scores [24]. Nevertheless, in practical situations, decision makers tend to express their preferences in discrete language terms, and managers are not very confident in subjective decision-making [25]. To design a more practical evaluation method, it is necessary to integrate the experience, knowledge or opinions from multiple decision-makers [26]. Fuzzy Multiple Attribute Group Decision Making (FMAGDM) method can coordinate the different opinions expressed by the decision-makers in fuzzy evaluation language, and then find a most acceptable alternative for the whole group [27]. The determination of evaluation attribute weight and decision-maker weight plays an important role in the FMAGDM framework. The method of determination directly affects the final evaluation result [28]. It is difficult in eliciting appropriate attribute weights from human experts [29]. In extant literature, the most prevailing methods to calculate attributes’ weights are: Delphi method [30], Analytic Hierarchy Process [31], Network Analysis [32], Entropy Method [33], Simos Procedure [34], Single Objective Optimization Model [35,36,37], Multi-Objective Optimization Model [38]. Govindan et al. have designed a decision-making framework for determining compromise group ranking according to the alternatives’ ranked by each decision maker participated in evaluation activity. The significant advantage of this framework is that it does not need the weight information of decision-makers [34]. Thus, the framework is used as a reference by the designed methods for ranking M-medical APPs in this paper.

## 3. Mobile Medical Service for Public

We use Internet search, questionnaire interview and other ways to collect 20 public-oriented mobile medical services in the Chinese market and focus on 10 well-known M-medical APPs. Their name and affiliated company information are shown in Table 1.

It is not always the patients who download and use the APP. To some extent, it is the person who care about their health or have health needs, usually called users. These mobile medical companies help countless users access to full-time and full-range medical and health services conveniently. Through the comprehensive analysis of the function modules of these 10 APPs, we summarized the user-oriented medical and health services of China mobile medical applications. M-medical APP provides users with one-stop health and medical services including 7*24-h online inquiry, consultation, interrogation, registration, drug purchase and other medical services, where consultation is the most important service for users.

### 3.1. Information Inquiry

Using M-mobile APPs, users can browse or understand health information independently, such as how to develop healthy living habits. Especially, during public health emergency, users can get timely information on the progress and protection of the epidemic. At the same time, users with medical needs can quickly query the schedule, charging and user evaluation of the corresponding hospital or doctor.

### 3.2. Consult with Doctor

For some mild or chronic diseases, patients need not to go to hospital immediately. They use M-mobile APPs to communicate with experts in the form of graphics, voice or video, so as to obtain professional advice or treatment programs. Some patients with common diseases can be diagnosed online. Different ways of communication correspond to different costs. Usually, video consultation is more expensive.

### 3.3. Registration and Appointment

After consulting doctors online, patients with more serious illnesses may need to be treated in offline hospitals. In this case, based on the M-mobile APPs, users can directly register online and appoint experts. Most of APPs registration fees are similar to offline registration fees, while the registration fees of few are significantly higher than that of offline. There is a significant difference in the cost of appointing experts of different levels or popularity, but it is generally more expensive.

### 3.4. Electronic Prescription

After fully mastering the patient’s condition, the consulted doctor issues an electronic prescription for the patient on the APP. According to the e-prescription, patients can not only buy drugs in the APP’s health mall, but also buy drugs in offline pharmacies or hospitals, which may be owned by mobile medical enterprises. The drugs can be directly delivered to the patients. It is no doubt that APP that can form electronic prescriptions or sell drugs to patients must be qualified.

### 3.5. Other Services

Other services of M-mobile APPs include two parts: one is free services such as health monitoring and health management; the other is charging services such as private doctors and disease management.

## 4. Preliminaries

In this part, a few fundamental concepts of the multiplicative linguistic evaluation scale, uncertain multiplicative linguistic variable and the superiority are introduced. We give the definition that the distance between any two UMLVs.

**Definition** **1.** *The scale of language evaluation is the reference scale for decision-makers to carry out qualitative evaluation. In the real situation, the decision-makers usually adopt the qualitative evaluation alternatives such as “good”, “not bad” and “general”, which are called as linguistic terms*

$$s_{\mu_{1}},s_{\mu_{2}},s_{\mu_{3}},s_{\mu_{4}},\cdots ,s_{\mu_{i}},\cdots ,s_{\mu_{t}}\in S,\mu_{t}\in Q^{+},$$

*where $\mu_{i}<\mu_{i+1},i=1,2,\cdots ,t$, and S is the language term set or language evaluation scale. When S meets two conditions, S is called as Multiplicative Linguistic Evaluation Scale, abbreviated as MLES: (a) if $\mu_{1}<\mu_{2}$, then $s_{\mu_{1}}<s_{\mu_{2}}$. (b) Reciprocal operator exists $neg(\mu_{3})=\mu_{4}$, and $\mu_{3}\times \mu_{4}=c$, in which c is constant. Taking $s_{c}$ as the boundary, the distance between the adjoining subscripts of the right part of $s_{c}$ is a constant, while that of the left part of $s_{c}$ increases with the increase of the subscript value [39].*

**Definition** **2.** *Supposed $s_{\mu_{1}},s_{\mu_{2}}\in S$, and λ≥0, the basic operational laws between two multiplicative linguistic terms $s_{\mu_{1}},s_{\mu_{2}}$ are defined [39]: (a) $s_{u_{1}}⊗s_{u_{2}}=s_{u_{1}\times u_{2}}$; (b) ${\left(s_{\mu_{1}}\right)}^{\lambda }=s_{\mu_{1}^{\lambda }}$.*

**Definition** **3.** *Let ${\tilde{s}}_{\alpha_{i}}=[s_{lo_{i}},s_{up_{i}}]$ be Uncertain Multiplicative Linguistic Variable, noted as UMLV simply. Here, $s_{lo},s_{up}\in S$, $\alpha_{i},i\in N^{+}$, $s_{lo},s_{up}$ given by the decision maker, represent the upper and lower limits of ${\tilde{s}}_{\alpha }$, respectively. Furtherly, let $\tilde{S}=\{{\tilde{s}}_{\alpha_{i}}\},i=1,2,\cdots ,n$ be UMLV set [39].*

**Definition** **4.** *For any two UMLVs ${\tilde{s}}_{\alpha_{1}}=[s_{lo1},s_{up1}],{\tilde{s}}_{\alpha_{2}}=[s_{lo2},s_{up2}]\in \tilde{S}$ and λ∈[0,1], the following some operational laws are defined [39]:*

(a) ${\tilde{s}}_{\alpha_{1}}⊗{\tilde{s}}_{\alpha_{2}}=[s_{lo1},s_{up1}]⊗[s_{lo2},s_{up2}]=[s_{lo1}⊗s_{lo2},s_{up1}⊗s_{up2}]=[s_{lo1\times lo2},s_{up1\times up2}]$;
(b) ${\tilde{s}}_{\alpha_{1}}^{\lambda }=[s_{lo_{1}}^{\lambda },s_{up_{1}}^{\lambda }]=[s_{lo_{1}^{\lambda }},s_{up_{1}^{\lambda }}]$.

**Definition** **5.** *Supposing ${\tilde{s}}_{\alpha_{1}},{\tilde{s}}_{\alpha_{2}},{\tilde{s}}_{\alpha_{3}}\in \tilde{S}$, and $\lambda ,\lambda_{1},\lambda_{2}\geq 0$, then they have the following operational properties [39].*

(a) ${\tilde{s}}_{\alpha_{1}}⊗{\tilde{s}}_{\alpha_{2}}={\tilde{s}}_{\alpha_{2}}⊗{\tilde{s}}_{\alpha_{1}}$;
(b) ${\left({\tilde{s}}_{\alpha_{1}}⊗{\tilde{s}}_{\alpha_{2}}\right)}^{\lambda }={\tilde{s}}_{\alpha_{1}}^{\lambda }⊗{\tilde{s}}_{\alpha_{2}}^{\lambda }$;
(c) ${\tilde{s}}_{\alpha_{3}}^{\lambda_{1}}⊗{\tilde{s}}_{\alpha_{3}}^{\lambda_{2}}={\tilde{s}}_{\alpha_{3}}^{\lambda_{1}+\lambda_{2}}$.

**Definition** **6.** *Let $p({\tilde{s}}_{\alpha_{1}}\geq {\tilde{s}}_{\alpha_{2}})$ be the probability of the event ${\tilde{s}}_{\alpha_{1}}\geq {\tilde{s}}_{\alpha_{2}}$. The probability is determined by the relative size of the total span of ${\tilde{s}}_{\alpha_{1}},{\tilde{s}}_{\alpha_{2}}$ and the sum of their own length. $p({\tilde{s}}_{\alpha_{1}}\geq {\tilde{s}}_{\alpha_{2}})$ is defined as follows [39]:*

$$p({\tilde{s}}_{\alpha_{1}}\geq {\tilde{s}}_{\alpha_{2}})=\min(\max(\frac{up1-lo2}{\left(up1-lo1)+(up2-lo2\right)},0),1).$$

*$p({\tilde{s}}_{\alpha_{1}}\geq {\tilde{s}}_{\alpha_{2}})$ can also be regarded as the superiority that ${\tilde{s}}_{\alpha_{1}}$ is over ${\tilde{s}}_{\alpha_{2}}$. It has the following properties:*

(a) $0\leq p({\tilde{s}}_{\alpha_{1}}\geq {\tilde{s}}_{\alpha_{2}})\leq 1$;
(b) *If and only if up2≤lo1, then $p({\tilde{s}}_{\alpha_{1}}\geq {\tilde{s}}_{\alpha_{2}})=1$;*
(c) *If and only if up1≤lo2, then $p({\tilde{s}}_{\alpha_{1}}\geq {\tilde{s}}_{\alpha_{2}})=0$;*
(d) *$p({\tilde{s}}_{\alpha_{1}}\geq {\tilde{s}}_{\alpha_{2}})+p({\tilde{s}}_{\alpha_{1}}\geq {\tilde{s}}_{\alpha_{2}})=1$, specially $p({\tilde{s}}_{\alpha_{1}}\geq {\tilde{s}}_{\alpha_{1}})=0.5$;*
(e) *If and only if up1+lo1≥up2+lo2, then $p({\tilde{s}}_{\alpha_{1}}\geq {\tilde{s}}_{\alpha_{2}})\geq 0.5$; If and only if up1+lo1=up2+lo2, then $p({\tilde{s}}_{\alpha_{1}}\geq {\tilde{s}}_{\alpha_{2}})=0.5$;*
(f) *If $p({\tilde{s}}_{\alpha_{1}}\geq {\tilde{s}}_{\alpha_{2}})\geq 0.5$ and $p({\tilde{s}}_{\alpha_{2}}\geq {\tilde{s}}_{\alpha_{3}})\geq 0.5$, then $p({\tilde{s}}_{\alpha_{1}}\geq {\tilde{s}}_{\alpha_{3}})\geq 0.5$.*

**Definition** **7.** *Let $dd({\tilde{s}}_{\alpha_{1}},{\tilde{s}}_{\alpha_{2}})$ be the distance between any two UMLVs ${\tilde{s}}_{\alpha_{1}}=[s_{lo1},s_{up1}],{\tilde{s}}_{\alpha_{2}}=[s_{lo2},s_{up2}]\in \tilde{S}$. $dd({\tilde{s}}_{\alpha_{1}},{\tilde{s}}_{\alpha_{2}})$ is computed by:*

$$dd({\tilde{s}}_{\alpha_{1}},{\tilde{s}}_{\alpha_{2}})=\frac{\left(up1/lo1)\times (up2/lo2\right)}{\mu_{t}}.$$

## 5. Uncertain Multiplicative Linguistic Decision Method

In this section, the process of individual ranking and group ranking is described in detail, and a general algorithm for group evaluation is formed.

The general fuzzy multi-attribute group decision-making problem is described as: there are *n* alternatives, *m* evaluation attributes and *h* decision-makers. The alternative set, evaluation attribute set, decision-maker set and decision group set are expressed as $X=\left\{X_{1},X_{2},\cdots ,X_{i},\cdots ,X_{n}\right\}$, $Y=\left\{Y_{1},Y_{2},\cdots ,Y_{j},\cdots ,Y_{m}\right\}$, $D=\left\{D_{1},D_{2},\cdots ,D_{k},\cdots ,D_{h}\right\}$. Simultaneously, $w_{j}^{k}\left(j=1,2,\cdots ,m;k=1,2\cdots ,h\right)$ represents the weight corresponding to the *j*-th attribute under preferences of *k*-th decision maker. The attribute weight vector of under *k*-th decision maker is recorded as $w^{k}=\{w_{1}^{k},w_{2}^{k},\cdots ,w_{j}^{k},\cdots ,w_{m}^{k}\}$. Generally, the individual preference is implied in individual language evaluation information.

### 5.1. Individual Alternatives Ranking

#### 5.1.1. Individual Decision Matrix

Referring to a given MLES, the *k*-th decision-maker’s uncertainty language information of the *j*-th attribute on the *i*-th alternative is noted as ${\tilde{s}}_{\alpha_{i,j,k}}$. Then, the *k*-th decision-maker’s evaluation matrix ${\tilde{A}}_{k}={\left\{{\tilde{s}}_{\alpha_{i,j,k}}\right\}}_{n\times m}$ is constructed as follows:

$${\tilde{A}}_{k}=\begin{array}{cc} & \begin{array}{cccccc} Y_{1} & Y_{2} & \cdots & Y_{j} & \cdots & Y_{m} \end{array}\\ \begin{array}{c} X_{1}\\ X_{2}\\ \vdots \\ \begin{array}{c} X_{i}\\ \begin{array}{c} \vdots \\ X_{n} \end{array} \end{array} \end{array} & \left(\begin{array}{cccc} \begin{array}{c} {\tilde{s}}_{\alpha_{1,1,k}}\\ {\tilde{s}}_{\alpha_{2,1,k}}\\ \vdots \\ \begin{array}{c} {\tilde{s}}_{\alpha_{i,1,k}}\\ \begin{array}{c} \vdots \\ {\tilde{s}}_{\alpha_{n,1,k}} \end{array} \end{array} \end{array} & \begin{array}{c} {\tilde{s}}_{\alpha_{1,2,k}}\\ {\tilde{s}}_{\alpha_{2,2,k}}\\ \vdots \\ \begin{array}{c} {\tilde{s}}_{\alpha_{i,2,k}}\\ \begin{array}{c} \vdots \\ {\tilde{s}}_{\alpha_{n,2,k}} \end{array} \end{array} \end{array} & \begin{array}{c} \cdots \\ \cdots \\ \cdots \\ \begin{array}{c} \cdots \\ \begin{array}{c} \cdots \\ \cdots \end{array} \end{array} \end{array} & \begin{array}{cc} \begin{array}{c} {\tilde{s}}_{\alpha_{1,j,k}}\\ {\tilde{s}}_{\alpha_{2,j,k}}\\ \vdots \\ \begin{array}{c} {\tilde{s}}_{\alpha_{i,j,k}}\\ \begin{array}{c} \vdots \\ {\tilde{s}}_{\alpha_{n,j,k}} \end{array} \end{array} \end{array} & \begin{array}{cc} \begin{array}{c} \cdots \\ \cdots \\ \cdots \\ \begin{array}{c} \cdots \\ \begin{array}{c} \cdots \\ \cdots \end{array} \end{array} \end{array} & \begin{array}{c} {\tilde{s}}_{\alpha_{1,m,k}}\\ {\tilde{s}}_{\alpha_{2,m,k}}\\ \vdots \\ \begin{array}{c} {\tilde{s}}_{\alpha_{i,m,k}}\\ \begin{array}{c} \vdots \\ {\tilde{s}}_{\alpha_{n,m,k}} \end{array} \end{array} \end{array} \end{array} \end{array} \end{array}\right) \end{array}.$$

The larger the evaluation value of some attributes is, the more favorable for the superiority of alternatives is. They are called positive attributes or benefit attributes. On the contrary, they are called negative attributes or cost attributes. For the cost attributes, we use the reciprocal operator to normalize the language evaluation information. For example, $\left[s_{1/4},s_{1/3}\right]$ is normalized as $\left[s_{3},s_{4}\right]$. We defined that ${\tilde{D}}_{k}$ is the result of normalized ${\tilde{A}}_{k}$.

#### 5.1.2. Individual Attributes’ Weights

From the perspective of information theory, if the evaluation value of alternatives under a certain attribute is similar, then the attribute is difficult to distinguish alternatives significantly, so the attribute should be given a smaller weight value [37].

According to the definition of $dd({\tilde{s}}_{\alpha_{1}},{\tilde{s}}_{\alpha_{2}})$, the total distance $d_{ij}(w^{k})$ between $X_{i}$ and other alternatives under $Y_{j}$ is obtained as follows:

(1) $$d_{ij}^{k}(w_{j}^{k})=\sum_{v\neq i,v=1}^{n}dd({\tilde{s}}_{\alpha_{i,j,k}},{\tilde{s}}_{\alpha_{v,j,k}})w_{j}^{k},j=1,2,\cdots ,m,k=1,2,\cdots ,h.$$

Furtherly, the total distance $d(w^{k})$ among alternatives under each evaluation attribute is represented by:

(2) $$\begin{array}{ll} d(w_{j}^{k}) & =\sum_{i=1}^{n}\sum_{j=1}^{m}d_{ij}\\ & =\sum_{i=1}^{n}\sum_{j=1}^{m}\sum_{v\neq i,v=1}^{n}dd({\tilde{s}}_{\alpha_{i,j,k}},{\tilde{s}}_{\alpha_{v,j,k}})w_{j}^{k} \end{array}$$

Therefore, the optimal attribute weight should meet the following single objective optimization model that maximize the differences among alternatives.

(3) $$\begin{array}{c} Max\;d(w^{k})=\sum_{i=1}^{n}\sum_{j=1}^{m}\sum_{v\neq i,v=1}^{n}dd({\tilde{s}}_{\alpha_{i,j,k}},{\tilde{s}}_{\alpha_{v,j,k}})w_{j}^{k}\\ s.t.\\ \left\{ \begin{array}{l} \sum_{j=1}^{m}{\left(w_{j}^{k}\right)}^{2}=1\\ w_{j}^{k}\geq 0 \end{array}\right. \end{array}$$

We introduce Lagrange parameter λ to transform the optimization model into Lagrange function $L(w^{k},\lambda )$.

(4) $$L(w^{k},\lambda )=d(w^{k})+\frac{1}{2}\lambda (\sum_{j=1}^{m}{\left(w_{j}^{k}\right)}^{2}-1).$$

According to the extremum theorem of continuous function, the best attribute weight ${\hat{w}}^{k}$ must satisfy the following necessary conditions.

(5) $$\left\{ \begin{array}{l} \frac{\partial L({\hat{w}}^{k},\lambda )}{\partial w_{j}^{k}}=\sum_{i=1}^{n}\sum_{v\neq i,v=1}^{n}dd({\tilde{s}}_{\alpha_{i,j,k}},{\tilde{s}}_{\alpha_{v,j,k}})=0,j=1,2,\cdots ,m\\ \frac{\partial L({\hat{w}}^{k},\lambda )}{\partial \lambda }=\sum_{j=1}^{m}{\left(w_{j}^{k}\right)}^{2}-1=0 \end{array}\right..$$

The solution ${\hat{w}}^{k}$ is as follows:

(6) $${\hat{w}}_{j}^{k}=\frac{\sum_{i=1}^{n}\sum_{v\neq i,v=1}^{n}dd({\tilde{s}}_{\alpha_{i,j,k}},{\tilde{s}}_{\alpha_{v,j,k}})}{\sqrt{\sum_{j=1}^{m}{\left(\sum_{i=1}^{n}\sum_{v\neq i,v=1}^{n}dd({\tilde{s}}_{\alpha_{i,j,k}},{\tilde{s}}_{\alpha_{v,j,k}})\right)}^{2}}},j=1,2,\cdots ,m.$$

Therefore, ${\hat{w}}^{k}$ is the extremum point and also the optimal point, because the $L({\hat{w}}^{k},\lambda )$ is a continuous differentiable convex function in feasible region. We normalize w as follows:

(7) $${\bar{w}}_{j}^{k}=\frac{\sum_{i=1}^{n}\sum_{v\neq i,v=1}^{n}dd({\tilde{s}}_{\alpha_{i,j,k}},{\tilde{s}}_{\alpha_{v,j,k}})}{\sum_{j=1}^{m}\sum_{i=1}^{n}\sum_{v\neq i,v=1}^{n}dd({\tilde{s}}_{\alpha_{i,j,k}},{\tilde{s}}_{\alpha_{v,j,k}})},j=1,2,\cdots ,m.$$

#### 5.1.3. Net Flow of Alternatives

**Definition** **8.** *The whole superiority between alternatives require a comprehensive comparison of the superiority of each attribute. Let $P_{k}(X_{i}>X_{v})$ be the probability that $X_{i}$ is superior to $X_{v}$ in whole for ${\bar{D}}_{k}$. $P(X_{i}>X_{v})$ is defined as follows [34]:*

(8) $$P_{k}(X_{i}>X_{v})=\sum_{j=1}^{m}w_{j}^{k}p_{j}(X_{i}>X_{v}).$$

**Definition** **9.** *Let $H_{k}^{+}(X_{v})$, $H_{k}^{-}(X_{v})$ and $H_{k}(X_{v})$ be positive flow, negative flow and net flow of $X_{v}$ for k-th decision-maker, respectively. Their expressions are as follows [34]:*

(9) $$H_{k}(X_{v})=H_{k}^{+}(X_{v})-H_{k}^{-}(X_{v}),v=1,2,\cdots ,n,$$

$$H_{k}^{+}(X_{v})=\frac{1}{n}\sum_{i=1}^{n}P_{k}(X_{v}>X_{i}),$$

$$H_{k}^{-}(X_{v})=\frac{1}{n}\sum_{i=1}^{n}P_{k}(X_{i}>X_{v}).$$

**Definition** **10.** *Let $T^{k}$ be the ranking of k-th decision-maker for all alternatives. For any two alternatives $X_{i}$ and $X_{v}$, their priority relationship judgment rules are as follows [34]:*

(a) *if $H_{k}(X_{i})>H_{k}(X_{v})$, $X_{i}$ is strictly superior to $X_{v}$, noted as $X_{i}≻X_{t}$;*
(b) *if $H_{k}(X_{i})∼H_{k}(X_{v})$, $X_{i}$ does not differ with $X_{v}$, noted as $X_{i}∼X_{t}$.*

### 5.2. Group Compromise Ranking

Individual ranking only represents own preference for alternatives and is an important basis for final group decision-making. Group compromise ranking refers to the alternatives ranking with the smaller total distance from the ranking given by each decision maker [34], which is represented by T. It can reflect the tendency of group selection.

**Definition** **11.** *Let $\sigma (T_{iv}^{k},T_{iv})$ be the distance between individual and group ranking on any two alternatives $X_{i}$ and $X_{v}$ [34]. $\sigma (T_{iv}^{k},T_{iv})$ is defined as the following Table 2:*

Then, the optimization model of minimizing the total distance among rankings is constructed. The decision variable is the priority relationship between any two alternatives $X_{i}$ and $X_{v}$ in group ranking, which is 0–1 variable. If $X_{i}≻X_{v}$ is true in optimal group ranking, $X_{iv}=1$; otherwise, $X_{iv}=0$. If $X_{i}∼X_{v}$ is true in optimal group ranking, $y_{iv}=1$; otherwise, $y_{iv}=0$.

(10) $$\begin{array}{l} \min\sum_{v=2}^{n}\sum_{i=1}^{v-1}\sum_{k=1}^{h}x_{iv}\sigma (T_{iv}^{k},X_{i}≻X_{v})+x_{vi}\sigma (T_{iv}^{k},X_{v}≻X_{i})+y_{iv}\sigma (T_{iv}^{k},X_{i}∼X_{v})\\ s.t.\\ \;\left\{ \begin{array}{l} 0\leq x_{iv}+x_{vi}<2\\ x_{iv}+x_{vi}+y_{iv}=1\\ \left\{ \begin{array}{l} x_{iv}\geq x_{ir}+x_{rv}-1.5\\ y_{iv}\geq y_{ir}+y_{rv}-1.5 \end{array}\right.,r=1,2,\cdots ,n\;and\;r\neq i\neq v\\ x_{iv},y_{iv}\in \{0,1\} \end{array}\right. \end{array}$$

In the optimal model, condition 1 limits $X_{i}≻X_{v}$ and $X_{v}≻X_{i}$ cannot occur at the same time. Condition 2 limits that only one case among $X_{i}≻X_{v}$, $X_{v}≻X_{i}$ and $X_{v}∼X_{i}$ can be true. Condition 3 makes strict priority relation and strict equivalence relation satisfy transitivity.

### 5.3. UMLDM Algorithm

Firstly, the alternatives, evaluation attributes, decision-makers and MLES are given. Secondly, the evaluation information of each decision-maker is collected to form the corresponding evaluation matrix. Thirdly, an optimization model is constructed to maximize the difference between alternatives, which is transformed into an unconstrained Lagrange function, and the preference attribute weights of each decision-maker is calculated. Then, alternatives’ positive flow, negative flow and net flow under decision makers’ evaluate are determined. At the same time, according to the value of net flow, the individual ranking is calculated. Finally, we minimize the total distance between individual rankings so as to obtain the group compromise ranking. The algorithm is named Uncertain Multiplicative Linguistic Decision Method based on Group Compromise Ranking Framework, abbreviated as UMLDM. The pseudocode of UMLDM is shown in Table 3.

From Table 2, it can be seen that the algorithm is composed of three loops, and correspondingly basic operation execution times are $O(h\times m\times n^{2})$, $O(h\times n^{2})$ and $O(h\times n^{2})$, respectively. Therefore, the time complexity of the algorithm is $O(h\times m\times n^{2})$.

## 6. Empirical Analysis

The proposed group evaluation algorithm is used to rank 10 well-known M-medical APPs, according to the experts’ uncertain preference information in the form of UMLVs collected by questionnaire.

### 6.1. The Assessment Indicator System

Before evaluating the alternatives, it is necessary to conduct a scientific and effective assessment indicator system. In the application of FMAGDM, the assessment criteria are usually determined by literature analysis and expert group discussion [7,21,40].

Users concerns various attributes of M-medical APPs. We discussed with 100 users of M-medical APPs to construct an applicable evaluation system. According to common indicators of published studies on APP evaluation [7,20,21,41], and users’ special needs for mobile medical services, we finally determined the assessment indicator system. The system consists of 8 evaluation indicators, where response time and price are cost attributes.

Professionalism ($Y_{1}$): It means the professional level of mainstream applications such as online consultation, chronic disease management and other medical services. The operating company behind the M-medical APP has a mature and professional team, including medical experts, technical personnel, especially its own medical team, offline clinics, pharmacies and other physical facilities.

Response time ($Y_{2}$): Response time indirectly affects user satisfaction. It reflects the average waiting time of users from consulting experts to getting responses. Some M-medical APPs lack of expert resources, low professional quality of doctors and imperfect supervision system, which leads to long application response time.

User-friendly ($Y_{3}$): The interface of M-medical APP shall be simple, beautiful and the functional modules shall be clear. It includes simplifying the operation process of the target user, providing multiple consultation methods and other friendly settings.

Price ($Y_{4}$): Users need to pay for medical services on the App. On the one hand, users must pay a fixed fee when purchasing drugs and devices on the App. on the other hand, users should pay different fees according to the professional level, consulting time and consulting methods when online consulting.

Additional services ($Y_{5}$). Additional services refer to free non-medical services provided by APP for users, such as drug distribution and free physical examination.

Security ($Y_{6}$): The personal information, disease information, consultation record and health monitoring data uploaded by users through M-medical APP are all private data. The application platform should adopt technical approaches and improve the confidentiality of operating system to protect the user’s privacy data.

User scale ($Y_{7}$): The user scale refers to the number of active users on the APP, which shows the comprehensive performance and popularity of the APP to a certain extent.

Reliability ($Y_{8}$): The capital, potential and reputation of the company to which the mobile medical APP belongs affect the reliability of the APP. In the early stage of mobile medical business, APP revenue is very limited, and the company has enough capital flow to maintain the normal operation of APP.

### 6.2. Questionnaire Design

Before evaluating activity, researcher ought to combine the characteristics of specific decision-making problems and the appropriate scale of language evaluation to design questionnaire and collect assessment information. Experts use evaluation language to express their intuitive perception of APP. These languages are ambiguous and uncertain. Considering that MLs can retain the original evaluation information to the greatest extent. Non-uniform MLES to $\mu_{t}=5$ is employed, the MLES is noted as $S_{1}$ in this paper.

$$\begin{array}{l} S_{1}=\{s_{1/5}=extreme\;low,s_{1/4}=very\;low,s_{1/3}=slightly\;low,s_{1/2}=low,s_{1}=medium,\\ s_{2}=high,s_{3}=slightly\;high,s_{4}=very\;high,s_{5}=extreme\;high\} \end{array}$$

**Figure 4:**
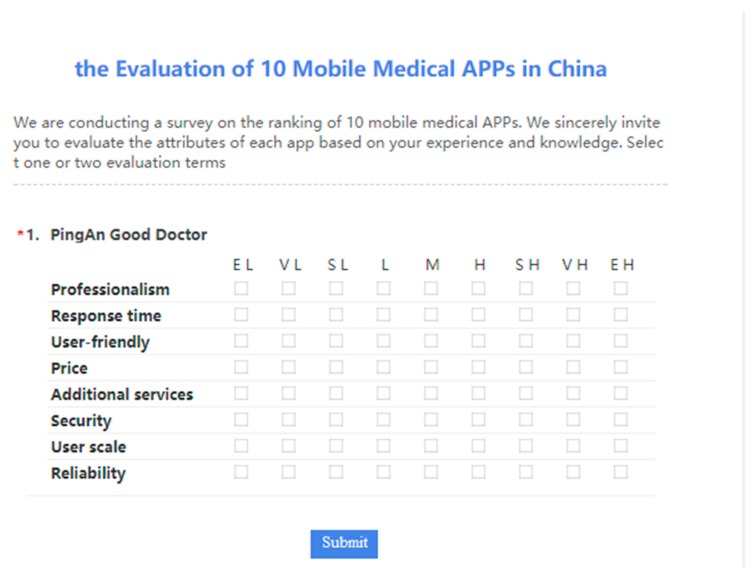
Some topics in the designed questionnaire.

Based on the given evaluation system and language measure scale, we design an evaluation questionnaire for 10 M-medical APPs as Table 1 shown, noted by $X_{1}\cdots X_{10}$ correspondingly. They large number of users in the current market and have their own characteristics and advantages, for example, $X_{1}$ has friendly interface and performance. Then, we push the questionnaire to the mobile phones of 5 decision-makers. Some topics of the designed questionnaire are shown in Figure 4. By collecting the questionnaire, we can get the language evaluation information of 5 experts, see Appendix A.

### 6.3. Assessment Process

Step1: we normalized the original language evaluation matrix of experts to form a normalization individual evaluation matrix. $D_{1}$ just is taken as an example in the paper, it is shown as Table 4.

Step 2: According to the Pseudocodes 3–4, the attributes of the weights under 5 experts’ assessment are calculated in the Table 5. It can be seen from the Table that experts 1 and 5 both attach importance to the professionalism of M-medical APPs, and the remaining three decision-makers are more inclined to the user-friendly, additional services and user scale of APP respectively.

Step 3: for 5 participants, the calculation result of positive flow $H_{k}^{+}(X_{v})$, negative flow $H_{k}^{-}(X_{v})$ and net flow $H_{k}(X_{v})$ of 5 alternatives are shown in the Table 6 and Table 7.

Step 4: Based on net flow of each mobile medical APP, 10 APPs Rankings of 5 decision makers are given in Table 8.

Step 5: we only consider the case where there is a priority relationship between APPs. By calculating the 0-1 optimal model, we can obtain $X_{iv}$ in Table 9.

Finally, the groups compromise ranking of 10 APPs is

$$X_{7}≻X_{5}≻X_{4}≻X_{3}≻X_{1}≻X_{2}≻X_{9}≻X_{6}≻X_{8}≻X_{10}.$$

**Figure 5:**
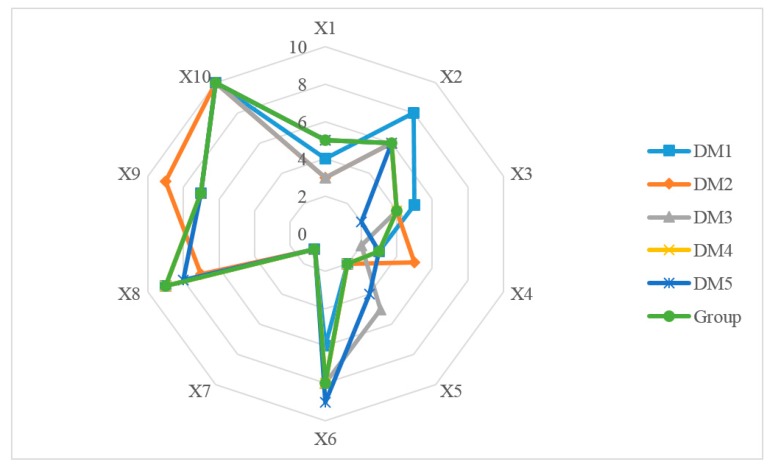
The individual ranking and group ranking for 10 M-medical APPs.

The total distance between group and individual ranking is 144. Therefore, the expert group is more inclined to the health 160 mobile medical application that has been highly recognized by the National Health Planning Commission, the Health Administration and other health administrative departments. 5 experts’ evolution and groups compromise assessment on 10 M-medical APPs of China are represented in Figure 5. It indicates that there are significant differences among experts’ preference for 10 given APPs, and the ultimate APP ranking coincides with the personal ranking of decision-maker 4.

## 7. Comparison Analysis

**Figure 6:**
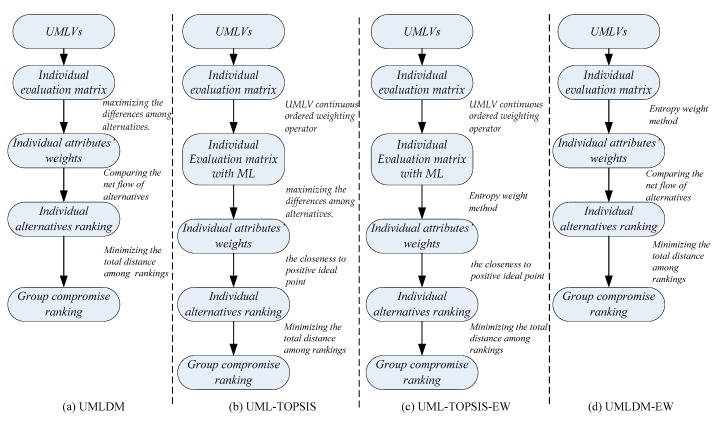
The procedure of 4 similar algorithms based on group compromise framework.

Entropy Weight (EW) is also a commonly used method to determine the weight from the perspective of information theory [33]. To verify the effectiveness and superiority of proposed method, the ranking of the alternatives calculated by UML-TOPSIS, UML-TOPSIS-EW and UMLDM-EW are compared with that of UMLDM (see Figure 6a). Similar to UMLDM, TOPSIS [7,21] is utilized for individual alternatives ranking, while other processes remain unchanged, which is named ULM-TOPSIS, see Figure 6b. Both ULM-TOPSIS-EW (see Figure 6c) and UMLDM-EW (see Figure 6d) are group decision-making algorithms, in which the corresponding attribute weight of each decision-maker is determined through the entropy weight method, according to ULM-TOPSIS and UMLDM, respectively. Other than that, we analyze the sensitivity of evaluation results to attribute weights calculated by two methods (Single objective optimization and entropy weight method).

### 7.1. UML-TOPSIS

#### 7.1.1. Deterministic ML Evaluation Matrix

**Definition** **12.** *Information integration operator can integrate personal evaluation information into group decision preference. Let $G_{\varphi (y)}$ be continuous ordered weighting operator. The definition is as follows [41]:*

$$G_{\varphi (y)}([a,b])=b{\left(\frac{a}{b}\right)}^{\int_{0}^{1}\frac{d\varphi (y)}{dy}ydy}=a^{\left(1-\int_{0}^{1}\varphi (y)dy\right)}b^{\int_{0}^{1}\varphi (y)d},$$

*where [a,b] is interval number, and φ(y) is BUM function, which is set as y/2.*

**Definition** **13.** *Let $ULG_{\varphi (y)}$ be UMLV continuous ordered weighting operator on φ(y). Supposed ${\tilde{s}}_{\alpha }=[s_{lo},s_{up}]\in \tilde{S}$, the following operational law is defined [41]:*

$$ULG_{\varphi }([{\tilde{s}}_{lo},{\tilde{s}}_{up}])={\tilde{s}}_{en},\;en=G_{\varphi }([I({\tilde{s}}_{lo}),I({\tilde{s}}_{up})])=G_{\varphi }([lo,up]),$$

*here, **I** is the subscript function.*

The individual assessment information matrix based on UML is transformed into the deterministic ML evaluation matrix by $ULG_{\varphi (y)}$ operator. Five experts’ deterministic ML evaluation matrix, noted as, ${\bar{D}}_{k},\;k=1,2,3,4,5$, respectively, are calculated in Appendix B.

#### 7.1.2. Individual Alternatives Ranking Based on TOPSIS

**Definition** **14.** *Let $SF_{i}^{k}=(s_{\alpha_{i,1,k}},s_{\alpha_{i,2,k}},\cdots ,s_{\alpha_{i,m,k}})$ be an attribute vector representing alternative $X_{i}$ for decision maker $D_{k}$. $PSF_{i}$, $NSF_{i}$ is positive ideal point and negative ideal point of $SF_{i}^{k}$, respectively. In this paper, according to utilized MLES, we determine $PSF_{i}$ and $NSF_{i}$ as following:*

$$PSF_{i}=(\underset{1\times m}{\underset{︸}{5,\cdots ,5}}),\;NSF_{i}=(\underset{1\times m}{\underset{︸}{1/5,\cdots ,1/5}}).$$

**Definition** **15.** *If $s_{\mu_{1}},s_{\mu_{2}}$ are any two multiplicative linguistic variables $s_{\mu_{1}},s_{\mu_{2}}\in S$, their deviation $MED(s_{\mu_{1}},s_{\mu_{2}})$ is defined as:*

$$MED(s_{\mu_{1}},s_{\mu_{2}})=\frac{\mu_{2}}{\mu_{1}}.$$

**Definition** **16.** *Let $MMED(SF_{i}^{k},SF_{v}^{k})$ be comprehensive deviation between any two alternative attribute vectors $SF_{i}^{k},SF_{v}^{k}$ of $D_{k}$. Expression of $MMED(s_{\alpha_{1}},s_{\alpha_{2}})$ is shown as following:*

$$MMED(SF_{i}^{k},SF_{v}^{k})=\sum_{j=1}^{m}w_{j}^{k}MED(SF_{i}^{k},SF_{v}^{k})+\sum_{j=1}^{m}w_{j}^{k}MED(SF_{v}^{k},SF_{i}^{k}),$$

*where $w_{j}(j=1,2,\cdots ,m)$ is calculated in the same way as UMLDM algorithm. Obviously, the sequence of MLVs is considered into $MED(s_{\mu_{1}},s_{\mu_{2}})$, while that do not need to be cared in $MMED(s_{\alpha_{1}},s_{\alpha_{2}})$.*

**Definition** **17.** *Let $Z_{k}(X_{i})$ represent the closeness to positive ideal point under $D_{k}$ assessing on $X_{i}$. It is calculated by:*

$$z_{k}(X_{i})=\frac{MMED(SF_{i}^{k},NSF_{i})}{\underset{v}{\max}MMED(SF_{v}^{k},NSF_{v})}-\frac{MMED(SF_{i}^{k},PSF_{i})}{\underset{v}{\min}MMED(SF_{v}^{k},PSF_{v})},$$

*where i,v=1,2,⋯,n and k=1,2,⋯,K. The greater the closeness of $X_{i}$ is, the decision-maker is more inclined to choose it, that is to say, $X_{i}$ is more advantageous in alternatives.*

**Figure 7:**
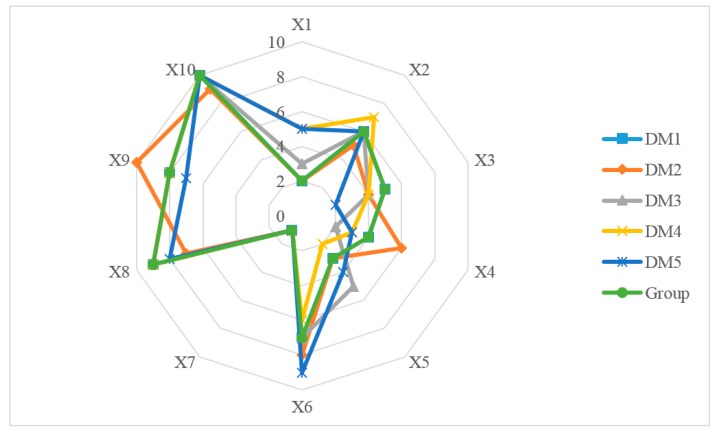
The individual ranking and group ranking for 10 mobile medical APPs based on UML-TOPSIS.

In this section, we calculate the ranking of 10 M-medical APPs for each expert based on from definitions 14 to 16, shown in Figure 7. The comprehensive deviation from attribute vector of each APPs to positive ideal point and negative ideal point, and alternative’s closeness to positive ideal point are given in Appendix C. Finally, according to Equation (10) and Table 2, the group compromise ranking of 10 M-medical APPs is obtained:

$$X_{7}≻X_{1}≻X_{5}≻X_{4}≻X_{3}≻X_{2}≻X_{6}≻X_{9}≻X_{8}≻X_{10},$$

and the total distance among group is calculated as 160. Additionally, the group is just same as the individual ranking of $D_{1}$. The time complexity of the UML-TOPSIS algorithm also is $O(h\times m\times n^{2})$.

### 7.2. UML-TOPSIS-EW

#### 7.2.1. Entropy Weight Method

**Definition** **18.** *Let $e_{j}^{k}$ be information entropy of $Y_{j}$ according to evaluation information of $D_{k}$, which is calculated by:*

$$e_{j}^{k}=-\varphi \sum_{i=1}^{n}O_{ij}^{k}\ln O_{ij}^{k},\;O_{ij}^{k}=\frac{\Gamma (s_{i,j,k})}{\sum_{i=1}^{n}\Gamma (s_{i,j,k})}$$

*where φ=1/lnn, $\Gamma (s_{i,j,k})$ is the subscript function of the multiplicative language $s_{i,j,k}$.*

Based on Definition 18, the $w_{j}^{k}$ is determined with the following expression:

$$w_{j}^{k}=\frac{1-e_{j}^{k}}{\sum_{j=1}^{m}1-e_{j}^{k}}.$$

Table 10 shows the attribute’s weight of 5 experts calculated by entropy method, which is significantly different from Table 5. In regard to individual preference, response time is most valued by $D_{1}$, additional services are emphasized by $D_{3}$, and price is most important for others.

#### 7.2.2. The Group Ranking of Mobile Medical APPs

**Figure 8:**
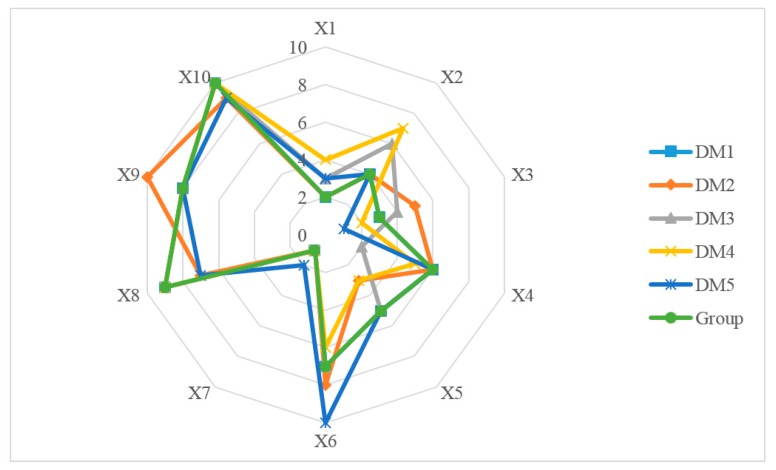
The individual ranking and group ranking for 10 mobile medical APPs based on UML-TOPSIS-EW.

We use the individual attribute weight in Table 9 to replace the that in UML-TOPSIS. The rules of individual ranking and group decision are the same as UML-TOPSIS. The comprehensive deviation from $X_{i}$ to $PSF_{i}$ and $NSF_{i}$, and each alternative closing to positive ideal APP are calculated in Appendix D. The individual ranking and for 10 M-medical APPs based on UML-TOPSIS-EW are presented in Figure 8. We obtain the group compromise ranking:

$$X_{7}≻X_{1}≻X_{3}≻X_{2}≻X_{5}≻X_{4}≻X_{6}≻X_{9}≻X_{8}≻X_{10}.$$

The tendency of the group is exactly the same as that of $D_{1}$. The corresponding total distance is 176. The time complexity of the algorithm is $\max(O(h\times m\times n),O(h\times n^{2}))$.

### 7.3. UMLDM-EW

Except for the calculation approach of alternative’s weight, the determination procedure of group ranking is completely consistent with UMLDM. Under expert’s language assessment information, we calculate the positive flow, the negative flow and the net flow of 10 M-medical APPs, as shown in Appendix E. The group compromise ranking is obtain:

$$X_{7}≻X_{4}≻X_{1}≻X_{3}≻X_{5}≻X_{2}≻X_{9}≻X_{6}≻X_{8}≻X_{10}.$$

**Figure 9:**
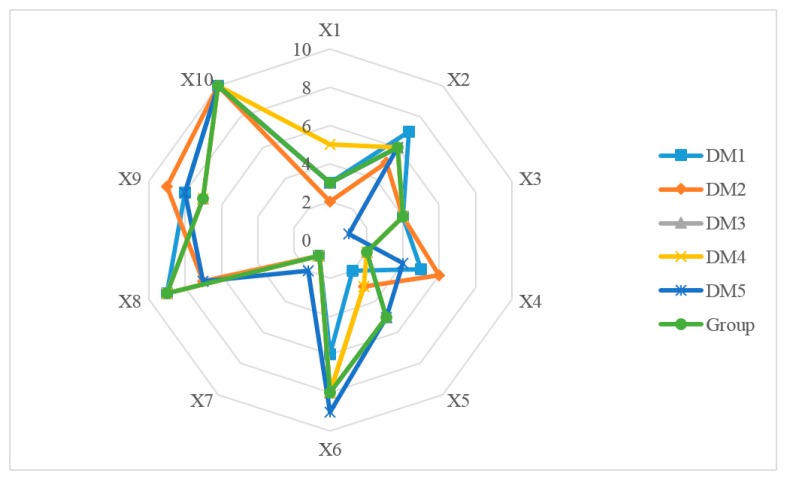
The individual ranking and group ranking for 10 mobile medical APPs based on UMLDM-EW.

It is consistent with the ranking of $D_{3}$. The total distance among rankings is 192. Figure 9 presents 5 expert’s rankings based on UMLDM-EW. The time complexity of the algorithm is

$$\max(O(h\times m\times n),O(h\times n^{2})).$$

### 7.4. Discussion

**Figure 10:**
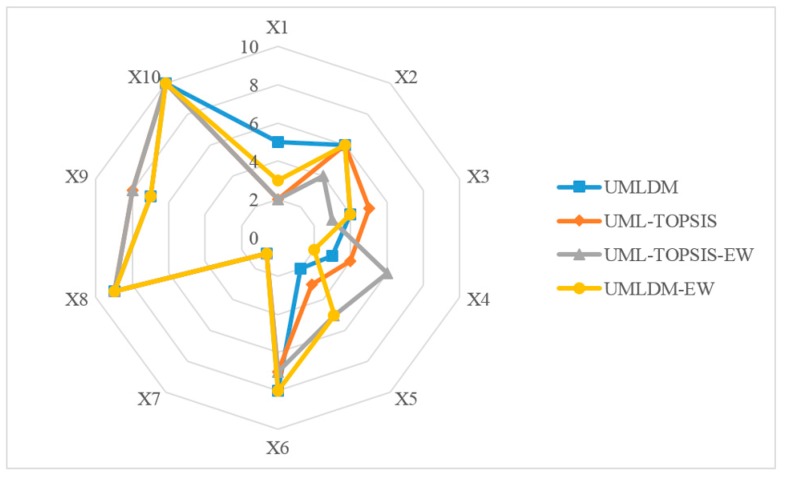
The group rankings of 10 mobile medical APPs determined by 4 FMAGDM based on group compromise ranking framework.

The group rankings of 10 M-medical APPs determine by 4 FMAGDM based on group compromise ranking framework are shown in Figure 10. Through comparison analysis, it concludes that (a) in general, the M-medical APPs ranking obtained by UMLDM, UML-TOPSIS, UML-TOPSIS-EW and UMLDM-EW are utterly similar provably, with Health 160 APP at the first, Micro-relationship APP at ninth and Access to Medical APP at the tenth; (b) the total distance among individual APP rankings increase in turn(144, 160, 176, 192) corresponding to 4 algorithms, where the algorithm has a larger total distance with applying entropy method to determine attribute weight, which means the group evaluation result is sensitive to the attribute weight; (c) the time complexity of UMLDM and UML-TOPSIS are $O(h\times m\times n^{2})$, while that of UML-TOPSIS-EW and UMLDM-EW $\max(O(h\times m\times n),O(h\times n^{2}))$.

After carried out comparison analysis, it indicates that the APP ranking calculated by UMLDM algorithm is highly consistent with that calculated by other 3 algorithms. The proposed algorithm can effectively solve FMAGDM problems based on individual language evaluation information. Although UMLDM algorithm is with higher time complexity, it can reduce the loss of evaluation information to some extent and has a better group compromise ranking.

## 8. Conclusions

With the improvement of living standards, residents pay more and more attention to their own health in China. Typically, they are eager to obtain high-quality medical resources conveniently and quickly and manage some chronic diseases by themselves. Mobile medicine developed with the advantages of mobile technology can not only meet people’s medical needs, but also effectively alleviate some medical dilemmas in China. In this environment, mobile medical applications with different characteristics are full of people’s daily life. M-medical APP can help patients reduce the decision-making cost, time cost and economic cost of consulting doctor and maintain health anywhere and anytime. Thus, it is vital for both users and relevant companies that how to choose the most suitable M-medical application for the masses in China’s existing market.

However, decision-makers are more inclined to use language terms to express themselves preference for alternatives in reality. Few studies have been carried out on evaluating mobile APPs from the perspective of UMLV, especially mobile medical APPs in China. To fill the gap, we firstly summarized the types of service provided by mobile medical APP for users. Then, a new multiple attributes group decision making algorithm considering uncertain multiplicative linguistic variable was designed, which is based on the group compromise ranking. The algorithm utilized UMLV to measure the decision-maker’s original preference information and the distance between any two UMLVs was defined, which contains three phases: the attribute weight calculated through maximizing the difference among alternatives; the APP ranking of each decision-maker determined based on its net flow; the group compromise ranking given by minimizing the total distance between individual ranking. It was used to rank 10 relatively well-known APPs based on the user-oriented assessment system including 8 indicators, and the calculation result was that Health 160 ≻ We Doctor ≻ Good Doctor Online ≻ DingXiang Doctor ≻ PingAn Good Doctor ≻ Alibab Health ≻ Medical Consultation Rapidly ≻ ChunYu Doctor ≻ Micro-relationship ≻ Access to Medical. Finally, the proposed algorithm is more effective and superior in evaluating mobile medical APPs and sensitive to the attribute weight, through the comparison and analysis of the ranking results determined by other similar 3 algorithms.

There are still some limitations in this study: (a) Uncertain multiplicative linguistic variables cannot measure the decision maker’s evaluation information completely. For this, intuitionistic fuzzy sets [42] and hesitant fuzzy sets [43] are usually utilized to collect real assessment information as much as possible. (b) The constructed evaluation system does not consider the information overlap effect between indicators. It is a better way that principal component analysis used to simplify the index system. (c) The designed evaluation framework can be further developed into a part of knowledge management system of smart hospital [44]. (d) The business model of mobile medical needs further development and innovation, so as to promote its widespread adoption among the public. These limitations are also the further research direction of this study.

## Figures and Tables

**Table 1:** 10 well-known M-medical APPs in the Chinese market.

NO.	Name	Affiliated Company
1	PingAn Good Doctor	Ping An Healthcare And Technology Company Limited
2	Alibab Health	Alibaba Group
3	DingXiang Doctor	Yinchuan Dingxiang Internet hospital Co., Ltd.
4	Good Doctor Online	Beijing Interactive Peak Technology Co., Ltd.
5	We Doctor	Guahao (Hangzhou) Technology Co., Ltd.
6	ChunYu Doctor	Beijing Spring Rain Software CO., Ltd.
7	Health 160	Shenzhen Ningyuan Technology Co., Ltd.
8	Micro-relationship	Hangzhou Choice Technology CO., Ltd.
9	Medical Consultation Rapidly	Hainan health cloud Internet hospital Co., Ltd.
10	Access to Medical	Daoyitong.com, Inc.

**Table 2:** Distance between different alternatives’ ranking.

	$X_{i}≻X_{v}$	$X_{i}∼X_{v}$	$X_{v}≻X_{i}$
$X_{i}≻X_{v}$	0	2	4
$X_{i}∼X_{v}$	2	0	4
$X_{v}≻X_{i}$	4	2	0

**Table 3:** The pseudocode of uncertain multiplicative linguistic decision method.

Input: individual evaluate matrix, and Table 2.
1. Using reciprocal operator to normalize ${\tilde{A}}_{k}$ and gaining matrix ${\bar{D}}_{k}$, where k=1,2,⋯,h; 2. let $dd_{1}=zeros(1,h)$ and $dd_{2}=zeros(m,h)$. They are used to record the total and *j*-th attribute corresponding to distance between alternatives under *k*-th decision maker respectively; 3. for *k*, from 1 to *h* for *j*, from 1 to *m* for *i*, from 1 to *n* for *v*, from 1 to *n* and v≠i $dd_{1}(1,k)=dd_{1}(1,k)+dd({\tilde{s}}_{i,j,k},{\tilde{s}}_{v,j,k}),\;dd_{2}(j,k)=dd_{2}(j,k)+dd({\tilde{s}}_{i,j,k},{\tilde{s}}_{v,j,k})$; end for *v* end for *i* end for *j* end for *k* 4. According to Equation (7), $\bar{w}$ is calculated by $dd_{2}/{\left(dd_{1}\right)}^{T}$; 5. for *k*, from 1 to *h* According to Equation (9), we get $H_{k}(X_{v})$ of any alternative $X_{v}$ and give ranking $T^{k}$; end for *k* 6. for *v*, from 2 to *n* for *i*, from 1 to *v*-1 for k, from 1 to *h* taking Table 1 and $T^{k}$ to Equation (10) and calculating $x_{iv}$ end for *k* end for *i* end for *v*
Output: group compromise ranking ***T***

**Table 4:** The normalized evaluation information matrix of $D_{1}$.

$D_{1}$	$Y_{1}$	$Y_{2}$	$Y_{3}$	$Y_{4}$	$Y_{5}$	$Y_{6}$	$Y_{7}$	$Y_{8}$
$X_{1}$	$\left[s_{2},s_{4}\right]$	$\left[s_{1/2},s_{1/3}\right]$	$\left[s_{1},s_{2}\right]$	$\left[s_{1/3},s_{1/2}\right]$	$\left[s_{2},s_{2}\right]$	$\left[s_{3},s_{4}\right]$	$\left[s_{3},s_{4}\right]$	$\left[s_{3},s_{3}\right]$
$X_{2}$	$\left[s_{1/3},s_{1/2}\right]$	$\left[s_{5},s_{5}\right]$	$\left[s_{1},s_{1}\right]$	$\left[s_{1/3},s_{1/3}\right]$	$\left[s_{1},s_{1}\right]$	$\left[s_{4},s_{4}\right]$	$\left[s_{1/2},s_{1}\right]$	$\left[s_{5},s_{5}\right]$
$X_{3}$	$\left[s_{4},s_{4}\right]$	$\left[s_{3},s_{5}\right]$	$\left[s_{1/2},s_{1}\right]$	$\left[s_{1/5},s_{1/3}\right]$	$\left[s_{3},s_{4}\right]$	$\left[s_{4},s_{5}\right]$	$\left[s_{1},s_{1}\right]$	$\left[s_{1},s_{2}\right]$
$X_{4}$	$\left[s_{4},s_{5}\right]$	$\left[s_{1/2},s_{1/2}\right]$	$\left[s_{4},s_{4}\right]$	$\left[s_{1/5},s_{1/5}\right]$	$\left[s_{2},s_{3}\right]$	$\left[s_{3},s_{3}\right]$	$\left[s_{1},s_{2}\right]$	$\left[s_{2},s_{3}\right]$
$X_{5}$	$\left[s_{3},s_{5}\right]$	$\left[s_{1},s_{2}\right]$	$\left[s_{2},s_{4}\right]$	$\left[s_{1/5},s_{1/4}\right]$	$\left[s_{2},s_{4}\right]$	$\left[s_{2},s_{2}\right]$	$\left[s_{3},s_{5}\right]$	$\left[s_{1},s_{1}\right]$
$X_{6}$	$\left[s_{2},s_{3}\right]$	$\left[s_{2},s_{3}\right]$	$\left[s_{1/3},s_{1}\right]$	$\left[s_{1/4},s_{1/3}\right]$	$\left[s_{1/3},s_{1/3}\right]$	$\left[s_{4},s_{5}\right]$	$\left[s_{1},s_{3}\right]$	$\left[s_{1/2},s_{1}\right]$
$X_{7}$	$\left[s_{3},s_{4}\right]$	$\left[s_{1/2},s_{1}\right]$	$\left[s_{3},s_{4}\right]$	$\left[s_{1/2},s_{1}\right]$	$\left[s_{1},s_{2}\right]$	$\left[s_{1},s_{1}\right]$	$\left[s_{4},s_{5}\right]$	$\left[s_{2},s_{2}\right]$
$X_{8}$	$\left[s_{2},s_{2}\right]$	$\left[s_{1/4},s_{1/3}\right]$	$\left[s_{1/4},s_{1}\right]$	$\left[s_{3},s_{3}\right]$	$\left[s_{1/3},s_{1/2}\right]$	$\left[s_{1/2},s_{1/2}\right]$	$\left[s_{2},s_{3}\right]$	$\left[s_{3},s_{5}\right]$
$X_{9}$	$\left[s_{1},s_{2}\right]$	$\left[s_{1},s_{1}\right]$	$\left[s_{2},s_{3}\right]$	$\left[s_{1/4},s_{1/4}\right]$	$\left[s_{1/2},s_{1}\right]$	$\left[s_{1},s_{2}\right]$	$\left[s_{3},s_{3}\right]$	$\left[s_{1/2},s_{1/2}\right]$
$X_{10}$	$\left[s_{1},s_{1}\right]$	$\left[s_{1/4},s_{1/4}\right]$	$\left[s_{2},s_{2}\right]$	$\left[s_{1},s_{2}\right]$	$\left[s_{1/5},s_{1/4}\right]$	$\left[s_{1/2},s_{1}\right]$	$\left[s_{1/4},s_{1/3}\right]$	$\left[s_{1/3},s_{1/2}\right]$

**Table 5:** The attribute’s weight of 5 experts.

	$D_{1}$	$D_{2}$	$D_{3}$	$D_{4}$	$D_{5}$
$Y_{1}$	**0.1826**	0.1342	0.1076	0.1336	**0.1551**
$Y_{2}$	0.0853	0.1361	0.1205	0.1056	0.1060
$Y_{3}$	0.1525	**0.1410**	0.1135	0.0895	0.1354
$Y_{4}$	0.1279	0.1314	0.1115	0.0929	0.0949
$Y_{5}$	0.1170	0.1130	**0.1493**	0.1668	0.1050
$Y_{6}$	0.0905	0.1186	0.1346	0.1178	0.1488
$Y_{7}$	0.1415	0.1056	0.1410	**0.1773**	0.1169
$Y_{8}$	0.1026	0.1202	0.1220	0.1164	0.1379

Remarks: the bold indicates the maximum value in the column.

**Table 6:** The positive, negative and net flow of $X_{v}$ for $D_{1},D_{2},D_{3}$.

	$D_{1}$			$D_{2}$			$D_{3}$		
	$H_{1}^{+}$	$H_{1}^{-}$	$H_{1}$	$H_{2}^{+}$	$H_{2}^{-}$	$H_{2}$	$H_{3}^{+}$	$H_{3}^{-}$	$H_{3}$
$X_{1}$	0.5936	0.3674	0.2261	0.5930	0.3314	0.2616	0.5963	0.3641	0.2322
$X_{2}$	0.3823	0.5586	−0.1763	0.5245	0.4531	0.0714	0.4806	0.4847	−0.0042
$X_{3}$	0.5540	0.4136	0.1404	0.5121	0.4139	0.0982	0.5875	0.3643	0.2232
$X_{4}$	0.6003	0.3455	0.2548	0.5240	0.4288	0.0952	0.6172	0.3455	0.2718
$X_{5}$	0.6403	0.3404	0.2999	0.6403	0.3337	0.3066	0.5224	0.4012	0.1212
$X_{6}$	0.4593	0.5138	−0.0545	0.3388	0.6351	−0.2964	0.3717	0.6161	−0.2444
$X_{7}$	0.6480	0.3327	0.3153	0.7132	0.2262	0.4870	0.6602	0.2617	0.3985
$X_{8}$	0.3054	0.6673	−0.3619	0.3332	0.6032	−0.2700	0.2983	0.6798	−0.3815
$X_{9}$	0.4037	0.5505	−0.1468	0.3138	0.6358	−0.3221	0.4244	0.5392	−0.1148
$X_{10}$	0.2304	0.7275	−0.4971	0.2658	0.6974	−0.4316	0.2057	0.7078	−0.5021

**Table 7:** The positive, negative and net flow of $X_{v}$ for $D_{4},D_{5}$.

	$D_{4}$			$D_{5}$		
	$H_{4}^{+}$	$H_{4}^{-}$	$H_{4}$	$H_{5}^{+}$	$H_{5}^{-}$	$H_{5}$
$X_{1}$	0.5155	0.4589	0.0566	0.5034	0.4453	0.0581
$X_{2}$	0.4983	0.4613	0.0370	0.5049	0.4623	0.0426
$X_{3}$	0.5449	0.4145	0.1304	0.6609	0.3055	0.3555
$X_{4}$	0.5955	0.3639	0.2316	0.6100	0.3239	0.2861
$X_{5}$	0.6205	0.3561	0.2645	0.5643	0.4156	0.1488
$X_{6}$	0.4167	0.5133	−0.0967	0.3384	0.6393	−0.3008
$X_{7}$	0.6286	0.3488	0.2798	0.6826	0.2941	0.3886
$X_{8}$	0.3832	0.5852	−0.2020	0.3490	0.6142	−0.2652
$X_{9}$	0.4579	0.5098	−0.0519	0.4213	0.5399	−0.1185
$X_{10}$	0.1482	0.7975	−0.6493	0.1630	0.7581	−0.5951

**Table 8:** APPs rankings of 5 exports.

Position	$D_{1}$	$D_{2}$	$D_{3}$	$D_{4}$	$D_{5}$
1	$X_{7}$	$X_{7}$	$X_{7}$	$X_{7}$	$X_{7}$
2	$X_{5}$	$X_{5}$	$X_{4}$	$X_{5}$	$X_{3}$
3	$X_{4}$	$X_{1}$	$X_{1}$	$X_{4}$	$X_{4}$
4	$X_{1}$	$X_{3}$	$X_{3}$	$X_{3}$	$X_{5}$
5	$X_{3}$	$X_{4}$	$X_{5}$	$X_{1}$	$X_{1}$
6	$X_{6}$	$X_{2}$	$X_{2}$	$X_{2}$	$X_{2}$
7	$X_{9}$	$X_{8}$	$X_{9}$	$X_{9}$	$X_{9}$
8	$X_{2}$	$X_{6}$	$X_{6}$	$X_{6}$	$X_{8}$
9	$X_{8}$	$X_{9}$	$X_{8}$	$X_{8}$	$X_{6}$
10	$X_{10}$	$X_{10}$	$X_{10}$	$X_{10}$	$X_{10}$

**Table 9:** The priority relationship between APPs.

$X_{iv}$	$X_{1}$	$X_{2}$	$X_{3}$	$X_{4}$	$X_{5}$	$X_{6}$	$X_{7}$	$X_{8}$	$X_{9}$	$X_{10}$
$X_{1}$	-	1	0	0	0	1	0	1	1	1
$X_{2}$	0	-	0	0	0	1	0	1	1	1
$X_{3}$	1	1	-	0	0	1	0	1	1	1
$X_{4}$	1	1	1	-	0	1	0	1	1	1
$X_{5}$	1	1	1	1	-	1	0	1	1	1
$X_{6}$	0	0	0	0	0	-	0	1	0	1
$X_{7}$	1	1	1	1	1	1	-	1	1	1
$X_{8}$	0	0	0	0	0	0	0	-	0	1
$X_{9}$	0	0	0	0	0	1	0	1	-	1
$X_{10}$	0	0	0	0	0	0	0	0	0	-

**Table 10:** The attribute’s weight of 5 experts calculated by entropy method.

	$D_{1}$	$D_{2}$	$D_{3}$	$D_{4}$	$D_{5}$
$Y_{1}$	0.0701	0.1181	0.1073	0.1281	0.1254
$Y_{2}$	**0.1606**	0.1259	0.1144	0.1076	0.1026
$Y_{3}$	0.1236	0.1144	0.1169	0.1187	0.0804
$Y_{4}$	0.1595	**0.1546**	0.1237	**0.1876**	**0.2057**
$Y_{5}$	0.1401	0.1429	**0.1453**	0.1203	0.1362
$Y_{6}$	0.1012	0.1292	0.1429	0.1452	0.1572
$Y_{7}$	0.1086	0.0974	0.1304	0.1128	0.0999
$Y_{8}$	0.1364	0.1175	0.1191	0.0797	0.0926

Remarks: The bold indicates the maximum value in the column.

## Appendix A

**Table A1:** Linguistic evaluation information matrix of $D_{1}$.

$D_{1}$	$Y_{1}$	$Y_{2}$	$Y_{3}$	$Y_{4}$	$Y_{5}$	$Y_{6}$	$Y_{7}$	$Y_{8}$
$X_{1}$	$\left[s_{2},s_{4}\right]$	$\left[s_{2},s_{3}\right]$	$\left[s_{1},s_{2}\right]$	$\left[s_{2},s_{3}\right]$	$\left[s_{2},s_{2}\right]$	$\left[s_{3},s_{4}\right]$	$\left[s_{3},s_{4}\right]$	$\left[s_{3},s_{3}\right]$
$X_{2}$	$\left[s_{1/3},s_{1/2}\right]$	$\left[s_{1/5},s_{1/5}\right]$	$\left[s_{1},s_{1}\right]$	$\left[s_{3},s_{3}\right]$	$\left[s_{1},s_{1}\right]$	$\left[s_{4},s_{4}\right]$	$\left[s_{1/2},s_{1}\right]$	$\left[s_{5},s_{5}\right]$
$X_{3}$	$\left[s_{4},s_{4}\right]$	$\left[s_{1/5},s_{1/3}\right]$	$\left[s_{1/2},s_{1}\right]$	$\left[s_{3},s_{5}\right]$	$\left[s_{3},s_{4}\right]$	$\left[s_{4},s_{5}\right]$	$\left[s_{1},s_{1}\right]$	$\left[s_{1},s_{2}\right]$
$X_{4}$	$\left[s_{4},s_{5}\right]$	$\left[s_{2},s_{2}\right]$	$\left[s_{4},s_{4}\right]$	$\left[s_{5},s_{5}\right]$	$\left[s_{2},s_{3}\right]$	$\left[s_{3},s_{3}\right]$	$\left[s_{1},s_{2}\right]$	$\left[s_{2},s_{3}\right]$
$X_{5}$	$\left[s_{3},s_{5}\right]$	$\left[s_{1/2},s_{1}\right]$	$\left[s_{2},s_{4}\right]$	$\left[s_{4},s_{5}\right]$	$\left[s_{2},s_{4}\right]$	$\left[s_{2},s_{2}\right]$	$\left[s_{3},s_{5}\right]$	$\left[s_{1},s_{1}\right]$
$X_{6}$	$\left[s_{2},s_{3}\right]$	$\left[s_{1/3},s_{1/2}\right]$	$\left[s_{1/3},s_{1}\right]$	$\left[s_{3},s_{4}\right]$	$\left[s_{1/3},s_{1/3}\right]$	$\left[s_{4},s_{5}\right]$	$\left[s_{1},s_{3}\right]$	$\left[s_{1/2},s_{1}\right]$
$X_{7}$	$\left[s_{3},s_{4}\right]$	$\left[s_{1},s_{2}\right]$	$\left[s_{3},s_{4}\right]$	$\left[s_{1},s_{2}\right]$	$\left[s_{1},s_{2}\right]$	$\left[s_{1},s_{1}\right]$	$\left[s_{4},s_{5}\right]$	$\left[s_{2},s_{2}\right]$
$X_{8}$	$\left[s_{2},s_{2}\right]$	$\left[s_{3},s_{4}\right]$	$\left[s_{1/4},s_{1}\right]$	$\left[s_{1/3},s_{1/3}\right]$	$\left[s_{1/3},s_{1/2}\right]$	$\left[s_{1/2},s_{1/2}\right]$	$\left[s_{2},s_{3}\right]$	$\left[s_{3},s_{5}\right]$
$X_{9}$	$\left[s_{1},s_{2}\right]$	$\left[s_{1},s_{1}\right]$	$\left[s_{2},s_{3}\right]$	$\left[s_{4},s_{4}\right]$	$\left[s_{1/2},s_{1}\right]$	$\left[s_{1},s_{2}\right]$	$\left[s_{3},s_{3}\right]$	$\left[s_{1/2},s_{1/2}\right]$
$X_{10}$	$\left[s_{1},s_{1}\right]$	$\left[s_{4},s_{4}\right]$	$\left[s_{2},s_{2}\right]$	$\left[s_{1/2},s_{1}\right]$	$\left[s_{1/5},s_{1/4}\right]$	$\left[s_{1/2},s_{1}\right]$	$\left[s_{1/4},s_{1/3}\right]$	$\left[s_{1/3},s_{1/2}\right]$

**Table A2:** Linguistic evaluation information matrix of $D_{2}$.

$D_{2}$	$Y_{1}$	$Y_{2}$	$Y_{3}$	$Y_{4}$	$Y_{5}$	$Y_{6}$	$Y_{7}$	$Y_{8}$
$X_{1}$	$\left[s_{1},s_{1}\right]$	$\left[s_{1},s_{1}\right]$	$\left[s_{1},s_{1}\right]$	$\left[s_{1/4},s_{1/2}\right]$	$\left[s_{2},s_{3}\right]$	$\left[s_{4},s_{4}\right]$	$\left[s_{3},s_{3}\right]$	$\left[s_{3},s_{3}\right]$
$X_{2}$	$\left[s_{1/3},s_{1/2}\right]$	$\left[s_{1/5},s_{1/3}\right]$	$\left[s_{1/2},s_{1}\right]$	$\left[s_{1/2},s_{1}\right]$	$\left[s_{1/2},s_{1}\right]$	$\left[s_{5},s_{5}\right]$	$\left[s_{1/3},s_{1/3}\right]$	$\left[s_{3},s_{4}\right]$
$X_{3}$	$\left[s_{2},s_{2}\right]$	$\left[s_{1/5},s_{1/5}\right]$	$\left[s_{1/3},s_{1/2}\right]$	$\left[s_{3},s_{3}\right]$	$\left[s_{3},s_{3}\right]$	$\left[s_{2},s_{3}\right]$	$\left[s_{1/2},s_{1/2}\right]$	$\left[s_{1},s_{1}\right]$
$X_{4}$	$\left[s_{4},s_{4}\right]$	$\left[s_{1/2},s_{1}\right]$	$\left[s_{1},s_{3}\right]$	$\left[s_{4},s_{5}\right]$	$\left[s_{1/3},s_{1/3}\right]$	$\left[s_{2},s_{2}\right]$	$\left[s_{1},s_{1}\right]$	$\left[s_{4},s_{5}\right]$
$X_{5}$	$\left[s_{3},s_{4}\right]$	$\left[s_{1/3},s_{1/2}\right]$	$\left[s_{3},s_{3}\right]$	$\left[s_{3},s_{4}\right]$	$\left[s_{3},s_{4}\right]$	$\left[s_{1},s_{1}\right]$	$\left[s_{4},s_{5}\right]$	$\left[s_{1/2},s_{1}\right]$
$X_{6}$	$\left[s_{1},s_{2}\right]$	$\left[s_{1},s_{2}\right]$	$\left[s_{1/2},s_{1/2}\right]$	$\left[s_{2},s_{3}\right]$	$\left[s_{1/4},s_{1/3}\right]$	$\left[s_{1/2},s_{1}\right]$	$\left[s_{1},s_{2}\right]$	$\left[s_{1/2},s_{1/2}\right]$
$X_{7}$	$\left[s_{2},s_{4}\right]$	$\left[s_{1/2},s_{1/2}\right]$	$\left[s_{4},s_{5}\right]$	$\left[s_{1/2},s_{1/2}\right]$	$\left[s_{1},s_{1}\right]$	$\left[s_{1},s_{2}\right]$	$\left[s_{4},s_{4}\right]$	$\left[s_{4},s_{4}\right]$
$X_{8}$	$\left[s_{1/2},s_{1/2}\right]$	$\left[s_{2},s_{2}\right]$	$\left[s_{1/3},s_{1/3}\right]$	$\left[s_{1/4},s_{1/3}\right]$	$\left[s_{1/3},s_{1/2}\right]$	$\left[s_{1/4},s_{1/4}\right]$	$\left[s_{2},s_{2}\right]$	$\left[s_{1},s_{2}\right]$
$X_{9}$	$\left[s_{1/4},s_{1/2}\right]$	$\left[s_{1/5},s_{1/4}\right]$	$\left[s_{4},s_{4}\right]$	$\left[s_{4},s_{4}\right]$	$\left[s_{1/2},s_{1/2}\right]$	$\left[s_{1/2},s_{1/2}\right]$	$\left[s_{2},s_{3}\right]$	$\left[s_{1/3},s_{1/2}\right]$
$X_{10}$	$\left[s_{1/4},s_{1/4}\right]$	$\left[s_{2},s_{3}\right]$	$\left[s_{2},s_{3}\right]$	$\left[s_{1/5},s_{1/4}\right]$	$\left[s_{1/4},s_{1/4}\right]$	$\left[s_{1/3},s_{1/2}\right]$	$\left[s_{1/3},s_{1/2}\right]$	$\left[s_{1/3},s_{1/3}\right]$

**Table A3:** Linguistic evaluation information matrix of $D_{3}$.

$D_{3}$	$Y_{1}$	$Y_{2}$	$Y_{3}$	$Y_{4}$	$Y_{5}$	$Y_{6}$	$Y_{7}$	$Y_{8}$
$X_{1}$	$\left[s_{2},s_{3}\right]$	$\left[s_{1/3},s_{1/2}\right]$	$\left[s_{1/3},s_{1/2}\right]$	$\left[s_{1},s_{1}\right]$	$\left[s_{2},s_{2}\right]$	$\left[s_{3},s_{3}\right]$	$\left[s_{2},s_{3}\right]$	$\left[s_{4},s_{5}\right]$
$X_{2}$	$\left[s_{1/3},s_{1/2}\right]$	$\left[s_{1/2},s_{1}\right]$	$\left[s_{1/3},s_{1/3}\right]$	$\left[s_{1/2},s_{1/2}\right]$	$\left[s_{1},s_{2}\right]$	$\left[s_{3},s_{5}\right]$	$\left[s_{1/4},s_{1/3}\right]$	$\left[s_{5},s_{5}\right]$
$X_{3}$	$\left[s_{2},s_{2}\right]$	$\left[s_{1/4},s_{1/4}\right]$	$\left[s_{1},s_{1}\right]$	$\left[s_{1},s_{2}\right]$	$\left[s_{4},s_{5}\right]$	$\left[s_{4},s_{5}\right]$	$\left[s_{1},s_{1}\right]$	$\left[s_{1/2},s_{1}\right]$
$X_{4}$	$\left[s_{4},s_{5}\right]$	$\left[s_{1/2},s_{1/2}\right]$	$\left[s_{4},s_{5}\right]$	$\left[s_{3},s_{3}\right]$	$\left[s_{3},s_{4}\right]$	$\left[s_{1},s_{2}\right]$	$\left[s_{2},s_{2}\right]$	$\left[s_{2},s_{3}\right]$
$X_{5}$	$\left[s_{3},s_{3}\right]$	$\left[s_{2},s_{2}\right]$	$\left[s_{2},s_{3}\right]$	$\left[s_{2},s_{2}\right]$	$\left[s_{4},s_{4}\right]$	$\left[s_{1},s_{1}\right]$	$\left[s_{3},s_{3}\right]$	$\left[s_{1},s_{2}\right]$
$X_{6}$	$\left[s_{1},s_{2}\right]$	$\left[s_{1},s_{2}\right]$	$\left[s_{1},s_{2}\right]$	$\left[s_{1/2},s_{1}\right]$	$\left[s_{1/3},s_{1}\right]$	$\left[s_{1/2},s_{1}\right]$	$\left[s_{1/3},s_{1}\right]$	$\left[s_{1},s_{1}\right]$
$X_{7}$	$\left[s_{3},s_{4}\right]$	$\left[s_{1/3},s_{1/3}\right]$	$\left[s_{3},s_{3}\right]$	$\left[s_{1/3},s_{1/2}\right]$	$\left[s_{1},s_{1}\right]$	$\left[s_{2},s_{2}\right]$	$\left[s_{4},s_{4}\right]$	$\left[s_{2},s_{2}\right]$
$X_{8}$	$\left[s_{1},s_{1}\right]$	$\left[s_{2},s_{3}\right]$	$\left[s_{1/4},s_{1/3}\right]$	$\left[s_{1/3},s_{1/3}\right]$	$\left[s_{1/4},s_{1/3}\right]$	$\left[s_{1/5},s_{1/3}\right]$	$\left[s_{1/2},s_{1}\right]$	$\left[s_{3},s_{5}\right]$
$X_{9}$	$\left[s_{1/2},s_{1/2}\right]$	$\left[s_{1/4},s_{1/3}\right]$	$\left[s_{3},s_{4}\right]$	$\left[s_{3},s_{4}\right]$	$\left[s_{1/2},s_{1}\right]$	$\left[s_{1/3},s_{1/3}\right]$	$\left[s_{3},s_{5}\right]$	$\left[s_{1/2},s_{1/2}\right]$
$X_{10}$	$\left[s_{1/3},s_{1/3}\right]$	$\left[s_{3},s_{3}\right]$	$\left[s_{2},s_{2}\right]$	$\left[s_{1/4},s_{1/4}\right]$	$\left[s_{1/3},s_{1/3}\right]$	$\left[s_{1/3},s_{1/2}\right]$	$\left[s_{1/4},s_{1/4}\right]$	$\left[s_{1/3},s_{1/3}\right]$

**Table A4:** Linguistic evaluation information matrix of $D_{4}$.

$D_{4}$	$Y_{1}$	$Y_{2}$	$Y_{3}$	$Y_{4}$	$Y_{5}$	$Y_{6}$	$Y_{7}$	$Y_{8}$
$X_{1}$	$\left[s_{1},s_{2}\right]$	$\left[s_{1},s_{2}\right]$	$\left[s_{1/2},s_{1/2}\right]$	$\left[s_{1/2},s_{1}\right]$	$\left[s_{1},s_{1}\right]$	$\left[s_{1},s_{2}\right]$	$\left[s_{2},s_{3}\right]$	$\left[s_{2},s_{3}\right]$
$X_{2}$	$\left[s_{1/4},s_{1/2}\right]$	$\left[s_{1/5},s_{1/5}\right]$	$\left[s_{1/3},s_{1/3}\right]$	$\left[s_{2},s_{2}\right]$	$\left[s_{1},s_{2}\right]$	$\left[s_{3},s_{4}\right]$	$\left[s_{1/3},s_{1}\right]$	$\left[s_{5},s_{5}\right]$
$X_{3}$	$\left[s_{2},s_{4}\right]$	$\left[s_{1/3},s_{1/3}\right]$	$\left[s_{2},s_{2}\right]$	$\left[s_{3},s_{3}\right]$	$\left[s_{3},s_{5}\right]$	$\left[s_{4},s_{4}\right]$	$\left[s_{1/3},s_{1/2}\right]$	$\left[s_{1},s_{2}\right]$
$X_{4}$	$\left[s_{4},s_{5}\right]$	$\left[s_{1},s_{1}\right]$	$\left[s_{4},s_{4}\right]$	$\left[s_{5},s_{5}\right]$	$\left[s_{2},s_{5}\right]$	$\left[s_{2},s_{2}\right]$	$\left[s_{1/2},s_{1}\right]$	$\left[s_{3},s_{4}\right]$
$X_{5}$	$\left[s_{3},s_{4}\right]$	$\left[s_{1/2},s_{1}\right]$	$\left[s_{3},s_{4}\right]$	$\left[s_{4},s_{5}\right]$	$\left[s_{4},s_{5}\right]$	$\left[s_{1},s_{1}\right]$	$\left[s_{2},s_{5}\right]$	$\left[s_{2},s_{2}\right]$
$X_{6}$	$\left[s_{1},s_{1}\right]$	$\left[s_{1/2},s_{1/2}\right]$	$\left[s_{1},s_{2}\right]$	$\left[s_{1},s_{2}\right]$	$\left[s_{2},s_{2}\right]$	$\left[s_{1/3},s_{1/2}\right]$	$\left[s_{1},s_{1}\right]$	$\left[s_{1},s_{1}\right]$
$X_{7}$	$\left[s_{4},s_{4}\right]$	$\left[s_{1/5},s_{1/3}\right]$	$\left[s_{2},s_{3}\right]$	$\left[s_{1},s_{1}\right]$	$\left[s_{1/2},s_{1}\right]$	$\left[s_{1/2},s_{1}\right]$	$\left[s_{3},s_{5}\right]$	$\left[s_{2},s_{4}\right]$
$X_{8}$	$\left[s_{1/2},s_{1/2}\right]$	$\left[s_{2},s_{3}\right]$	$\left[s_{1/4},s_{1/4}\right]$	$\left[s_{1/4},s_{1/4}\right]$	$\left[s_{1/3},s_{1/2}\right]$	$\left[s_{1/4},s_{1/2}\right]$	$\left[s_{2},s_{4}\right]$	$\left[s_{4},s_{5}\right]$
$X_{9}$	$\left[s_{1/2},s_{1}\right]$	$\left[s_{1/5},s_{1/4}\right]$	$\left[s_{3},s_{3}\right]$	$\left[s_{3},s_{4}\right]$	$\left[s_{1/3},s_{1}\right]$	$\left[s_{1/2},s_{1/2}\right]$	$\left[s_{4},s_{5}\right]$	$\left[s_{1/2},s_{1/2}\right]$
$X_{10}$	$\left[s_{1/3},s_{1/2}\right]$	$\left[s_{2},s_{2}\right]$	$\left[s_{1/3},s_{1/2}\right]$	$\left[s_{1/3},s_{1/3}\right]$	$\left[s_{1/3},s_{1/3}\right]$	$\left[s_{1/4},s_{1/3}\right]$	$\left[s_{1/3},s_{1/3}\right]$	$\left[s_{1/2},s_{1}\right]$

**Table A5:** Linguistic evaluation information matrix of $D_{5}$.

$D_{5}$	$Y_{1}$	$Y_{2}$	$Y_{3}$	$Y_{4}$	$Y_{5}$	$Y_{6}$	$Y_{7}$	$Y_{8}$
$X_{1}$	$\left[s_{1},s_{1}\right]$	$\left[s_{1/3},s_{1/2}\right]$	$\left[s_{1/3},s_{1/3}\right]$	$\left[s_{1/2},s_{1}\right]$	$\left[s_{2},s_{2}\right]$	$\left[s_{3},s_{4}\right]$	$\left[s_{3},s_{3}\right]$	$\left[s_{1},s_{2}\right]$
$X_{2}$	$\left[s_{1/2},s_{1}\right]$	$\left[s_{1/3},s_{1/3}\right]$	$\left[s_{1},s_{2}\right]$	$\left[s_{1},s_{2}\right]$	$\left[s_{1},s_{1}\right]$	$\left[s_{4},s_{5}\right]$	$\left[s_{1/2},s_{1/2}\right]$	$\left[s_{2},s_{4}\right]$
$X_{3}$	$\left[s_{1},s_{3}\right]$	$\left[s_{1/4},s_{1/4}\right]$	$\left[s_{2},s_{2}\right]$	$\left[s_{1/2},s_{1/2}\right]$	$\left[s_{3},s_{5}\right]$	$\left[s_{2},s_{4}\right]$	$\left[s_{1},s_{2}\right]$	$\left[s_{2},s_{3}\right]$
$X_{4}$	$\left[s_{3},s_{3}\right]$	$\left[s_{1/2},s_{1}\right]$	$\left[s_{3},s_{3}\right]$	$\left[s_{4},s_{5}\right]$	$\left[s_{4},s_{4}\right]$	$\left[s_{2},s_{2}\right]$	$\left[s_{2},s_{2}\right]$	$\left[s_{3},s_{4}\right]$
$X_{5}$	$\left[s_{3},s_{4}\right]$	$\left[s_{1/2},s_{1/2}\right]$	$\left[s_{2},s_{3}\right]$	$\left[s_{3},s_{3}\right]$	$\left[s_{4},s_{5}\right]$	$\left[s_{1/2},s_{1}\right]$	$\left[s_{3},s_{4}\right]$	$\left[s_{1/2},s_{1}\right]$
$X_{6}$	$\left[s_{1},s_{2}\right]$	$\left[s_{2},s_{2}\right]$	$\left[s_{2},s_{4}\right]$	$\left[s_{2},s_{3}\right]$	$\left[s_{1},s_{2}\right]$	$\left[s_{1/4},s_{1/3}\right]$	$\left[s_{1},s_{1}\right]$	$\left[s_{1/3},s_{1}\right]$
$X_{7}$	$\left[s_{2},s_{3}\right]$	$\left[s_{1/4},s_{1/2}\right]$	$\left[s_{3},s_{4}\right]$	$\left[s_{1},s_{1}\right]$	$\left[s_{3},s_{4}\right]$	$\left[s_{1},s_{2}\right]$	$\left[s_{2},s_{3}\right]$	$\left[s_{3},s_{3}\right]$
$X_{8}$	$\left[s_{1/3},s_{1}\right]$	$\left[s_{1},s_{2}\right]$	$\left[s_{1},s_{1}\right]$	$\left[s_{1/4},s_{1/4}\right]$	$\left[s_{1/3},s_{1/2}\right]$	$\left[s_{1/3},s_{1/2}\right]$	$\left[s_{1},s_{3}\right]$	$\left[s_{2},s_{2}\right]$
$X_{9}$	$\left[s_{1/2},s_{1/2}\right]$	$\left[s_{1/4},s_{1/3}\right]$	$\left[s_{1},s_{4}\right]$	$\left[s_{4},s_{4}\right]$	$\left[s_{1/2},s_{1}\right]$	$\left[s_{1/3},s_{1}\right]$	$\left[s_{4},s_{5}\right]$	$\left[s_{1},s_{1}\right]$
$X_{10}$	$\left[s_{1/3},s_{1/3}\right]$	$\left[s_{3},s_{3}\right]$	$\left[s_{1/2},s_{1/2}\right]$	$\left[s_{1/4},s_{1/3}\right]$	$\left[s_{1/3},s_{1/3}\right]$	$\left[s_{1},s_{1}\right]$	$\left[s_{1/3},s_{1/2}\right]$	$\left[s_{1/2},s_{1/2}\right]$

## Appendix B

**Table A6:** Deterministic linguistic evaluation information matrix of $D_{1}$.

$D_{1}$	$Y_{1}$	$Y_{2}$	$Y_{3}$	$Y_{4}$	$Y_{5}$	$Y_{6}$	$Y_{7}$	$Y_{8}$
$X_{1}$	$s_{2.3784}$	$s_{0.5000}$	$s_{1.1892}$	$s_{0.3689}$	$s_{2.0000}$	$s_{3.2237}$	$s_{3.2237}$	$s_{3.0000}$
$X_{2}$	$s_{0.5217}$	$s_{2.2361}$	$s_{1.0000}$	$s_{0.3333}$	$s_{1.0000}$	$s_{4.0000}$	$s_{0.5946}$	$s_{5.0000}$
$X_{3}$	$s_{4.0000}$	$s_{3.4087}$	$s_{0.5946}$	$s_{0.2272}$	$s_{3.2237}$	$s_{4.2295}$	$s_{1.0000}$	$s_{1.1892}$
$X_{4}$	$s_{4.2295}$	$s_{0.5000}$	$s_{4.0000}$	$s_{0.2000}$	$s_{2.2134}$	$s_{3.0000}$	$s_{1.1892}$	$s_{2.2134}$
$X_{5}$	$s_{3.4087}$	$s_{1.1892}$	$s_{2.3784}$	$s_{0.2115}$	$s_{2.3784}$	$s_{2.0000}$	$s_{3.4087}$	$s_{1.0000}$
$X_{6}$	$s_{2.2134}$	$s_{2.2134}$	$s_{0.3333}$	$s_{1.4142}$	$s_{0.3333}$	$s_{4.2295}$	$s_{1.3161}$	$s_{0.5946}$
$X_{7}$	$s_{3.2237}$	$s_{0.5946}$	$s_{3.2237}$	$s_{0.5946}$	$s_{1.1892}$	$s_{1.0000}$	$s_{4.2295}$	$s_{2.0000}$
$X_{8}$	$s_{2.0000}$	$s_{0.2686}$	$s_{0.3536}$	$s_{1.7321}$	$s_{0.3689}$	$s_{0.5000}$	$s_{2.2134}$	$s_{3.4087}$
$X_{9}$	$s_{1.1892}$	$s_{1.0000}$	$s_{2.2134}$	$s_{0.2500}$	$s_{0.5946}$	$s_{1.1892}$	$s_{3.0000}$	$s_{0.5000}$
$X_{10}$	$s_{1.0000}$	$s_{0.2500}$	$s_{2.0000}$	$s_{1.1892}$	$s_{0.2115}$	$s_{0.5946}$	$s_{0.2686}$	$s_{0.3689}$

**Table A7:** Deterministic linguistic evaluation information matrix of $D_{2}$.

$D_{2}$	$Y_{1}$	$Y_{2}$	$Y_{3}$	$Y_{4}$	$Y_{5}$	$Y_{6}$	$Y_{7}$	$Y_{8}$
$X_{1}$	$s_{1.0000}$	$s_{1.0000}$	$s_{1.0000}$	$s_{2.3784}$	$s_{2.2134}$	$s_{4.0000}$	$s_{3.0000}$	$s_{3.0000}$
$X_{2}$	$s_{0.3689}$	$s_{3.4087}$	$s_{0.5946}$	$s_{1.1892}$	$s_{0.5946}$	$s_{5.0000}$	$s_{0.3333}$	$s_{3.2237}$
$X_{3}$	$s_{2.0000}$	$s_{5.0000}$	$s_{0.3689}$	$s_{0.3333}$	$s_{3.0000}$	$s_{2.2134}$	$s_{0.5000}$	$s_{1.0000}$
$X_{4}$	$s_{4.0000}$	$s_{1.1892}$	$s_{1.3161}$	$s_{0.2115}$	$s_{0.3333}$	$s_{2.0000}$	$s_{1.0000}$	$s_{4.2295}$
$X_{5}$	$s_{3.2237}$	$s_{2.2134}$	$s_{3.0000}$	$s_{0.2686}$	$s_{3.2237}$	$s_{1.0000}$	$s_{4.2295}$	$s_{0.5946}$
$X_{6}$	$s_{1.1892}$	$s_{0.5946}$	$s_{0.5000}$	$s_{0.3689}$	$s_{0.2686}$	$s_{0.5946}$	$s_{1.1892}$	$s_{0.5000}$
$X_{7}$	$s_{2.3784}$	$s_{2.0000}$	$s_{4.2295}$	$s_{2.0000}$	$s_{1.0000}$	$s_{1.1892}$	$s_{4.0000}$	$s_{4.0000}$
$X_{8}$	$s_{0.5000}$	$s_{0.5000}$	$s_{0.3333}$	$s_{3.2237}$	$s_{0.3689}$	$s_{0.2500}$	$s_{2.0000}$	$s_{1.1892}$
$X_{9}$	$s_{0.2973}$	$s_{0.2115}$	$s_{4.0000}$	$s_{0.2500}$	$s_{0.5000}$	$s_{0.5000}$	$s_{2.2134}$	$s_{0.3689}$
$X_{10}$	$s_{0.2500}$	$s_{0.3689}$	$s_{2.2134}$	$s_{4.2295}$	$s_{0.2500}$	$s_{0.3689}$	$s_{0.3689}$	$s_{0.3333}$

**Table A8:** Deterministic linguistic evaluation information matrix of $D_{3}$.

$D_{3}$	$Y_{1}$	$Y_{2}$	$Y_{3}$	$Y_{4}$	$Y_{5}$	$Y_{6}$	$Y_{7}$	$Y_{8}$
$X_{1}$	$s_{2.2134}$	$s_{2.2134}$	$s_{0.3689}$	$s_{1.0000}$	$s_{2.0000}$	$s_{3.0000}$	$s_{2.2134}$	$s_{4.2295}$
$X_{2}$	$s_{0.3689}$	$s_{1.1892}$	$s_{0.3333}$	$s_{2.0000}$	$s_{1.1892}$	$s_{3.4087}$	$s_{0.2686}$	$s_{5.0000}$
$X_{3}$	$s_{2.0000}$	$s_{4.0000}$	$s_{1.0000}$	$s_{0.5946}$	$s_{4.2295}$	$s_{4.2295}$	$s_{1.0000}$	$s_{0.5946}$
$X_{4}$	$s_{4.2295}$	$s_{2.0000}$	$s_{4.2295}$	$s_{0.3333}$	$s_{3.2237}$	$s_{1.1892}$	$s_{2.0000}$	$s_{2.2134}$
$X_{5}$	$s_{3.0000}$	$s_{0.5000}$	$s_{2.2134}$	$s_{0.5000}$	$s_{4.0000}$	$s_{1.0000}$	$s_{3.0000}$	$s_{1.1892}$
$X_{6}$	$s_{1.1892}$	$s_{0.5946}$	$s_{1.1892}$	$s_{1.1892}$	$s_{0.4387}$	$s_{0.5946}$	$s_{0.4387}$	$s_{1.0000}$
$X_{7}$	$s_{3.2237}$	$s_{3.0000}$	$s_{3.0000}$	$s_{2.2134}$	$s_{1.0000}$	$s_{2.0000}$	$s_{4.0000}$	$s_{2.0000}$
$X_{8}$	$s_{1.0000}$	$s_{0.3689}$	$s_{0.2686}$	$s_{3.0000}$	$s_{0.2686}$	$s_{0.2272}$	$s_{0.5946}$	$s_{3.4087}$
$X_{9}$	$s_{0.5000}$	$s_{3.2237}$	$s_{3.2237}$	$s_{0.2686}$	$s_{0.5946}$	$s_{0.3333}$	$s_{3.4087}$	$s_{0.5000}$
$X_{10}$	$s_{0.3333}$	$s_{0.3333}$	$s_{2.0000}$	$s_{4.0000}$	$s_{0.3333}$	$s_{0.3689}$	$s_{0.2500}$	$s_{0.3333}$

**Table A9:** Deterministic linguistic evaluation information matrix of $D_{4}$.

$D_{4}$	$Y_{1}$	$Y_{2}$	$Y_{3}$	$Y_{4}$	$Y_{5}$	$Y_{6}$	$Y_{7}$	$Y_{8}$
$X_{1}$	$s_{1.1892}$	$s_{0.5946}$	$s_{0.5000}$	$s_{1.1892}$	$s_{1.0000}$	$s_{1.1892}$	$s_{2.2134}$	$s_{2.2134}$
$X_{2}$	$s_{0.2973}$	$s_{5.0000}$	$s_{0.3333}$	$s_{0.5000}$	$s_{1.1892}$	$s_{3.2237}$	$s_{0.4387}$	$s_{5.0000}$
$X_{3}$	$s_{2.3784}$	$s_{3.0000}$	$s_{2.0000}$	$s_{0.3333}$	$s_{3.4087}$	$s_{4.0000}$	$s_{0.3689}$	$s_{1.1892}$
$X_{4}$	$s_{4.2295}$	$s_{1.0000}$	$s_{4.0000}$	$s_{0.2000}$	$s_{2.5149}$	$s_{2.0000}$	$s_{0.5946}$	$s_{3.2237}$
$X_{5}$	$s_{3.2237}$	$s_{1.1892}$	$s_{3.2237}$	$s_{0.2115}$	$s_{4.2295}$	$s_{1.0000}$	$s_{2.5149}$	$s_{2.0000}$
$X_{6}$	$s_{1.0000}$	$s_{2.0000}$	$s_{1.1892}$	$s_{0.5946}$	$s_{2.0000}$	$s_{0.3689}$	$s_{1.0000}$	$s_{1.0000}$
$X_{7}$	$s_{4.0000}$	$s_{3.4087}$	$s_{2.2134}$	$s_{1.0000}$	$s_{0.5946}$	$s_{0.5946}$	$s_{3.4087}$	$s_{2.3784}$
$X_{8}$	$s_{0.5000}$	$s_{0.3689}$	$s_{0.2500}$	$s_{4.0000}$	$s_{0.3689}$	$s_{0.2973}$	$s_{2.3784}$	$s_{4.2295}$
$X_{9}$	$s_{0.5946}$	$s_{4.2295}$	$s_{3.0000}$	$s_{0.2686}$	$s_{0.4387}$	$s_{0.5000}$	$s_{4.2295}$	$s_{0.5000}$
$X_{10}$	$s_{0.3689}$	$s_{0.5000}$	$s_{0.3689}$	$s_{3.0000}$	$s_{0.3333}$	$s_{0.2686}$	$s_{0.3333}$	$s_{0.5946}$

**Table A10:** Deterministic linguistic evaluation information matrix of $D_{5}$.

$D_{5}$	$Y_{1}$	$Y_{2}$	$Y_{3}$	$Y_{4}$	$Y_{5}$	$Y_{6}$	$Y_{7}$	$Y_{8}$
$X_{1}$	$s_{1.0000}$	$s_{2.2134}$	$s_{0.3333}$	$s_{1.1892}$	$s_{2.0000}$	$s_{3.2237}$	$s_{3.0000}$	$s_{1.1892}$
$X_{2}$	$s_{0.5946}$	$s_{3.0000}$	$s_{1.1892}$	$s_{0.5946}$	$s_{1.0000}$	$s_{4.2295}$	$s_{0.5000}$	$s_{2.3784}$
$X_{3}$	$s_{1.3161}$	$s_{4.0000}$	$s_{2.0000}$	$s_{2.0000}$	$s_{3.4087}$	$s_{2.3784}$	$s_{1.1892}$	$s_{2.2134}$
$X_{4}$	$s_{3.0000}$	$s_{1.1892}$	$s_{3.0000}$	$s_{0.2115}$	$s_{2.0000}$	$s_{2.0000}$	$s_{2.0000}$	$s_{3.2237}$
$X_{5}$	$s_{3.2237}$	$s_{2.0000}$	$s_{2.2134}$	$s_{0.3333}$	$s_{4.2295}$	$s_{0.5946}$	$s_{3.2237}$	$s_{0.5946}$
$X_{6}$	$s_{1.1892}$	$s_{0.5000}$	$s_{2.3784}$	$s_{0.3689}$	$s_{1.1892}$	$s_{0.2686}$	$s_{1.0000}$	$s_{0.4387}$
$X_{7}$	$s_{2.2134}$	$s_{2.3784}$	$s_{3.2237}$	$s_{1.0000}$	$s_{3.2237}$	$s_{1.1892}$	$s_{2.2134}$	$s_{3.0000}$
$X_{8}$	$s_{0.4387}$	$s_{0.5946}$	$s_{1.0000}$	$s_{4.0000}$	$s_{0.3689}$	$s_{0.3689}$	$s_{1.3161}$	$s_{2.0000}$
$X_{9}$	$s_{0.5000}$	$s_{3.2237}$	$s_{1.4142}$	$s_{0.2500}$	$s_{0.5946}$	$s_{0.4387}$	$s_{4.2295}$	$s_{1.0000}$
$X_{10}$	$s_{0.3333}$	$s_{0.3333}$	$s_{1.0000}$	$s_{3.2237}$	$s_{0.3333}$	$s_{1.0000}$	$s_{0.3689}$	$s_{0.5000}$

## Appendix C

**Table A11:** $MMED(SF_{i}^{k},NSF_{i})$, $MMED(SF_{i}^{k},PSF_{i})$ and $z_{k}(X_{i})$ for $D_{1},D_{2},D_{3}$ based on UML-TOPSIS.

	$D_{1}$			$D_{2}$			$D_{3}$		
	To P	To N	CD	To P	To N	CD	To P	To N	CD
$X_{1}$	4.4357	9.9766	−0.3652	3.1125	10.6268	−0.4486	3.6712	10.9434	−0.6792
$X_{2}$	6.6128	7.7878	−1.1408	6.5947	9.1814	−1.9732	7.5176	8.6398	−2.4828
$X_{3}$	5.8778	10.8230	−0.6857	6.4667	9.0956	−1.9278	3.9912	11.4568	−0.7733
$X_{4}$	5.7000	11.8820	−0.5483	7.0438	9.0031	−2.1692	3.6610	12.0332	−0.5885
$X_{5}$	5.1648	10.7894	−0.4949	5.1003	10.9639	−1.2298	4.3635	9.9497	−1.0497
$X_{6}$	6.6077	7.4690	−1.1663	9.6453	3.2298	−3.6682	7.4625	3.9827	−2.8288
$X_{7}$	3.6815	11.2019	−0.0572	2.4631	13.0378	0.0000	2.3731	12.6107	0.0000
$X_{8}$	7.5319	7.0754	−1.4504	9.6933	5.1560	−3.5400	11.5742	5.4372	−4.4461
$X_{9}$	6.7242	6.6248	−1.2689	12.4629	5.2963	−4.6537	8.2155	7.5668	−2.8619
$X_{10}$	11.0916	4.0772	−2.6697	12.1021	5.5135	−4.4905	12.5587	4.6220	−4.9256

**Table A12:** $MMED(SF_{i}^{k},NSF_{i})$, $MMED(SF_{i}^{k},PSF_{i})$ and $z_{k}(X_{i})$ for $D_{4},D_{5}$ based on UML-TOPSIS.

	$D_{4}$			$D_{5}$		
	To P	To N	CD	To P	To N	CD
$X_{1}$	4.7284	6.6700	−0.6528	4.7134	8.7596	−1.1297
$X_{2}$	7.6454	9.4113	−1.1716	5.0081	8.7419	−1.2471
$X_{3}$	5.3585	10.4424	−0.4962	2.5674	11.2234	−0.0807
$X_{4}$	5.4175	10.9160	−0.4713	4.1837	12.0676	−0.6470
$X_{5}$	4.4157	11.8336	−0.1360	4.9517	10.1729	−1.1064
$X_{6}$	5.5720	5.8871	−0.9360	8.6503	4.6838	−3.0172
$X_{7}$	3.8869	11.1806	−0.0552	2.5402	11.5732	−0.0410
$X_{8}$	9.4268	7.5514	−1.7871	7.6831	5.8474	−2.5401
$X_{9}$	7.5796	8.7996	−1.2064	7.4993	6.9717	−2.3746
$X_{10}$	12.5693	3.1465	−2.9679	10.6996	3.7832	−3.8987

Remarks: To P means the comprehensive deviation from $X_{i}$ to $PSF_{i}$; To N means the comprehensive deviation from $X_{i}$ to $NSF_{i}$; CD means the closeness to $PSF_{i}$ for $X_{i}$.

## Appendix D

**Table A13:** $MMED(SF_{i}^{k},NSF_{i})$, $MMED(SF_{i}^{k},PSF_{i})$ and $z_{k}(X_{i})$ for $D_{1},D_{2},D_{3}$ based on UML-TOPSIS-EW.

	$D_{1}$			$D_{2}$			$D_{3}$		
	To P	To N	CD	To P	To N	CD	To P	To N	CD
$X_{1}$	5.3377	9.0922	−0.3004	2.9595	11.0195	−0.2455	3.7410	10.8451	−0.7087
$X_{2}$	5.9179	9.3192	−0.4060	6.3708	9.3350	−1.6877	7.3652	8.7613	−2.4006
$X_{3}$	6.3259	10.4385	−0.3883	6.3822	9.2017	−1.7028	4.0303	11.4134	−0.7851
$X_{4}$	7.0814	9.7372	−0.6213	7.8588	8.5290	−2.3226	3.8283	11.9050	−0.6608
$X_{5}$	6.1986	8.9975	−0.4985	5.5065	10.5785	−1.2565	4.4384	9.7826	−1.0871
$X_{6}$	6.7184	7.3807	−0.7674	10.1255	3.0965	−3.6284	7.3638	4.0340	−2.7775
$X_{7}$	4.5564	9.0719	−0.1309	2.6111	12.4098	0.0000	2.3757	12.5230	0.0000
$X_{8}$	8.7274	6.5543	−1.2875	9.6544	5.3901	−3.2632	11.5886	5.5351	−4.4360
$X_{9}$	7.7157	5.7744	−1.1402	12.7315	4.7651	−4.4920	8.4820	7.3532	−2.9831
$X_{10}$	12.5738	3.5817	−2.4164	12.2012	5.7076	−4.2130	12.2823	4.8807	−4.7802

**Table A14:** $MMED(SF_{i}^{k},NSF_{i})$, $MMED(SF_{i}^{k},PSF_{i})$ and $z_{k}(X_{i})$ for $D_{4},D_{5}$ based on UML-TOPSIS-EW.

	$D_{4}$			$D_{5}$		
	To P	To N	CD	To P	To N	CD
$X_{1}$	5.0660	6.0891	−0.6528	4.0703	9.0638	−0.8569
$X_{2}$	8.0144	8.8434	−1.1087	5.3538	8.3572	−1.4357
$X_{3}$	5.7812	10.2735	−0.4251	2.4793	11.5480	0.0000
$X_{4}$	7.2021	10.3930	−0.7610	6.5954	10.7021	−1.7334
$X_{5}$	6.5623	10.3018	−0.6134	6.1349	9.5130	−1.6507
$X_{6}$	6.2171	5.4136	−0.9993	9.5635	4.0616	−3.5056
$X_{7}$	4.0897	10.3072	−0.0083	2.9044	10.5571	−0.2573
$X_{8}$	9.7516	7.8837	−1.6259	7.5388	7.2212	−2.4154
$X_{9}$	8.6474	7.9003	−1.3543	9.3302	6.1174	−3.2335
$X_{10}$	11.6029	4.3587	−2.4177	9.6517	5.3259	−3.4317

## Appendix E

**Table A15:** The positive, negative and net flow of $X_{v}$ for $D_{1},D_{2},D_{3}$ based on UMLDM-EW.

	$D_{1}$			$D_{2}$			$D_{3}$		
	$H_{1}^{+}$	$H_{1}^{-}$	$H_{1}$	$H_{2}^{+}$	$H_{2}^{-}$	$H_{2}$	$H_{3}^{+}$	$H_{3}^{-}$	$H_{3}$
$X_{1}$	0.5771	0.3631	0.2140	0.6137	0.3161	0.2976	0.5920	0.3668	0.2252
$X_{2}$	0.4343	0.4996	−0.0653	0.5399	0.4374	0.1025	0.4863	0.4777	0.0087
$X_{3}$	0.5790	0.4031	0.1759	0.5254	0.3990	0.1265	0.5859	0.3672	0.2187
$X_{4}$	0.5291	0.4003	0.1288	0.4970	0.4543	0.0427	0.6127	0.3504	0.2623
$X_{5}$	0.6010	0.3752	0.2258	0.6307	0.3450	0.2857	0.5170	0.4066	0.1103
$X_{6}$	0.4998	0.4739	0.0259	0.3354	0.6414	−0.3059	0.3755	0.6126	−0.2372
$X_{7}$	0.6171	0.3592	0.2579	0.6982	0.2380	0.4603	0.6602	0.2629	0.3972
$X_{8}$	0.3057	0.6771	−0.3714	0.3502	0.5913	−0.2411	0.3019	0.6750	−0.3731
$X_{9}$	0.4101	0.5334	−0.1233	0.2996	0.6462	−0.3466	0.4124	0.5506	−0.1382
$X_{10}$	0.2481	0.7165	−0.4683	0.2703	0.6919	−0.4216	0.2201	0.6942	−0.4741

**Table A16:** The positive, negative and net flow of $X_{v}$ for $D_{4},D_{5}$ based on UMLDM-EW.

	$D_{4}$			$D_{5}$		
	$H_{4}^{+}$	$H_{4}^{-}$	$H_{4}$	$H_{5}^{+}$	$H_{5}^{-}$	$H_{5}$
$X_{1}$	0.5313	0.4448	0.0864	0.5518	0.4040	0.1477
$X_{2}$	0.4874	0.4632	0.0241	0.5022	0.4640	0.0382
$X_{3}$	0.5469	0.3972	0.1498	0.6864	0.2748	0.4116
$X_{4}$	0.5522	0.3918	0.1604	0.5304	0.4097	0.1208
$X_{5}$	0.5656	0.4119	0.1537	0.5295	0.4397	0.0897
$X_{6}$	0.4254	0.5198	−0.0944	0.3212	0.6585	−0.3373
$X_{7}$	0.6229	0.3456	0.2773	0.6468	0.3233	0.3235
$X_{8}$	0.3889	0.5677	−0.1788	0.4039	0.5583	−0.1544
$X_{9}$	0.4397	0.5260	−0.0863	0.3711	0.5865	−0.2154
$X_{10}$	0.2274	0.7197	−0.4923	0.2531	0.6775	−0.4244
